# Immune activation during pregnancy exacerbates ASD-related alterations in *Shank3*-deficient mice

**DOI:** 10.1186/s13229-022-00532-3

**Published:** 2023-01-05

**Authors:** Ekaterina Atanasova, Andrea Pérez Arévalo, Ines Graf, Rong Zhang, Juergen Bockmann, Anne-Kathrin Lutz, Tobias M. Boeckers

**Affiliations:** 1 Institute for Anatomy and Cell Biology, Ulm University, Ulm, Germany; 2 Neuroscience Research Institute, Health Science Centre, Peking University, Peking, China; 3 Deutsches Zentrum für Neurodegenerative Erkrankungen (DZNE), Ulm Site, Ulm, Germany

**Keywords:** ASD, MIA, Poly I:C, Postsynaptic density, SHANK3, Two-hit

## Abstract

**Background:**

Autism spectrum disorder (ASD) is mainly characterized by deficits in social interaction and communication and repetitive behaviors. Known causes of ASD are mutations of certain risk genes like the postsynaptic protein SHANK3 and environmental factors including prenatal infections.

**Methods:**

To analyze the gene-environment interplay in ASD, we combined the *Shank3Δ11*−*/*− ASD mouse model with maternal immune activation (MIA) via an intraperitoneal injection of polyinosinic/polycytidylic acid (Poly I:C) on gestational day 12.5. The offspring of the injected dams was further analyzed for autistic-like behaviors and comorbidities followed by biochemical experiments with a focus on synaptic analysis.

**Results:**

We show that the two-hit mice exhibit excessive grooming and deficits in social behavior more prominently than the *Shank3Δ11*−*/*− mice. Interestingly, these behavioral changes were accompanied by an unexpected upregulation of postsynaptic density (PSD) proteins at excitatory synapses in striatum, hippocampus and prefrontal cortex.

**Limitations:**

We found several PSD proteins to be increased in the two-hit mice; however, we can only speculate about possible pathways behind the worsening of the autistic phenotype in those mice.

**Conclusions:**

With this study, we demonstrate that there is an interplay between genetic susceptibility and environmental factors defining the severity of ASD symptoms. Moreover, we show that a general misbalance of PSD proteins at excitatory synapses is linked to ASD symptoms, making this two-hit model a promising tool for the investigation of the complex pathophysiology of neurodevelopmental disorders.

**Supplementary Information:**

The online version contains supplementary material available at 10.1186/s13229-022-00532-3.

## Background

Autism spectrum disorders (ASDs) are complex neurodevelopmental disorders characterized by two main symptoms—(i) deficits in social interaction and communication and (ii) restricted and repetitive behaviors [1]. The term “spectrum” has been introduced to indicate the heterogeneity of symptoms seen in autistic people as well as the variety of causes. In addition, several comorbidities are associated with ASD including intellectual disability, language impairment and motor deficits. Regarding the causes of ASD, twin and family studies suggest a strong genetic contribution [2–4]. Interestingly among the autism risk genes are several genes coding for synaptic proteins like the SHANK family of postsynaptic scaffolding molecules [5, 6]. SHANKs are highly concentrated at postsynaptic densities of excitatory synapses and play a key role in synaptogenesis and synaptic plasticity, especially during postnatal brain development [7–9]. Thorough analyses of stratified patient cohorts revealed that there is a gradient of severity when it comes to the different members of this protein family, with *SHANK3* mutations being the most common (0.69% of ASD patients) and leading to the most severe phenotype [10]. Moreover, in the majority of cases, the heterogeneous disruption or deletion of *SHANK3* has been shown to lead to a syndromic form of autism, the Phelan–McDermid syndrome [11]*.* Over the years, several mouse models carrying mutations in the *Shank3* gene have been generated [12]. These knockout mice presented reduced interest for social interaction in the three-chamber test and free social interaction including abnormalities in the emission of ultrasonic vocalizations (USVs) [13–15]. The models also displayed repetitive self-grooming as well as specific synaptic abnormalities such as a reduction in the number of synapses [13, 15, 16].

While there is a significant genetic component in ASD, many studies indicate that certain environmental factors significantly contribute to the pathogenesis of ASD [17, 18]. A higher risk to develop ASD for dizygotic twins compared to non-twin siblings hints at a role for the maternal environment [19]. Epidemiological studies have associated the occurrence of a viral infection during the first and second trimester of pregnancy with an increased risk for the children to develop ASD [20, 21], while a weaker link was found for a bacterial infection [20]. Thus, maternal immune activation (MIA) mouse models have been created in order to study the mechanisms through which an early life infection might cause long-term behavioral defects. Polyinosinic/polycytidylic acid (Poly I:C) is commonly used to mimic immune activation by a viral infection. This synthetic dsRNA molecule interacts with toll-like receptor-3 (TLR-3) and increases the production of proinflammatory cytokines and chemokines in the embryonic brain [22, 23]. Behavioral abnormalities related to ASD like decreased social novelty recognition and communication have been found in the offspring of mice following an immune activation with Poly I:C, as well as increased repetitive behaviors [19, 24]. Moreover, these mouse models present reduced dendritic branching and dendritic spine density and altered synaptic transmission in the hippocampus [25–27].

Two-hit preclinical models are based on the hypothesis that multiple factors, genetic and environmental, converge and lead to the development of the adult pathology. Two-hit and even multiple-hit rodent models have been extensively used in schizophrenia research [28, 29]. Concerning autism, some studies have combined two environmental factors and have found a worsening of the autistic-like phenotype [30, 31]. However, only a few autism-related studies have combined environmental factors with genetic susceptibility [32, 33]. Interestingly, the application of valproic acid (VPA) and propionic acid (PPA) was found to induce alterations in the expression of SHANK proteins in those models [34].

To further elucidate the interplay of internal (genetic predisposition) and external factors (environment) related to Shank3-ASD, we combined a partial knockout mouse model *Shank3Δ11*−*/*− with MIA by intraperitoneal injection of Poly I:C on gestational day (GD) 12.5. With this two-hit model, we aim to contribute to the understanding of phenotypic severity in Shank3-ASD. Therefore, we investigated the core symptoms of autism as well as comorbidities by the means of a wide set of behavioral experiments. To link that behavior to the synapse as one of the central structures affected by SHANK3 deficiency, further biochemical analysis was conducted.

## Methods

### Generation of *Shank3* knockout mice

The *Shank3Δ11*−*/*− , + /− and + /+ mice were bred as described previously [16]. They were housed under constant temperature (22 ± 1 °C) and humidity (50%) conditions with a 12 h light/dark cycle and provided with food and water ad libitum. Using heterozygous mice for breeding, we derived wild-type, heterozygous and knockout littermates. PCR genotyping of *Shank3Δ11*−*/*− was performed using the following sets of oligonucleotide primers: for WT allele forward 5′-CAAGTTCATCGCTGTGAAGG-3′, for mutant allele forward 5′-CCTCTAGGCCTGCTAGCTGTT-3′ and for both WT and mutant allele reverse 5′-AAGAAGCCCCAGAAGTGACA-3′.

### Timed pregnancy

Mice were mated overnight with a mating regime of 1:1. The next day the male was removed from the cage and the female was weighed and the presence or absence of a vaginal plug was noted marking the onset of pregnancy or gestational day (GD) 0.5. Females were not disturbed, except for weekly cage cleaning, until GD11.5 when they were weighed again. As the presence of a vaginal plug is not a guarantee for pregnancy, an additional method (previously described by [35, 36]) was used to confirm the pregnancy. Mice that showed an increase in body weight from GD0.5 to GD11.5 of 20–30% were confirmed pregnant and were pseudo-randomly assigned to one of the two groups, Poly I:C or saline. The success rate of the breedings was 25%.

### Maternal administration of Poly I:C

The maternal immune activation (MIA) model was made by injecting polyinosinic/polycytidylic acid (Poly I:C) (Potassium salt; Sigma, P9582-5MG) as previously described [37]. Pregnant dams on GD12.5 received either a single intraperitoneal injection of Poly I:C or vehicle solution (NaCl 0.9%, Fresenius Kabi Germany GmbH, cat. # 04986179). The Poly I:C was dissolved in saline to obtain the desired dosage (20 mg/kg). The volume of injection was 8 ml/kg [38, 39]. The same volume of saline was injected into pregnant dams at the same time to prepare the control mice. The solution was freshly prepared each time before injection. The injection was made using a 1 ml syringe and a 27 G needle and in the middle of the belly in order to avoid hitting the embryos. The cap of the needle was cut and put back on making the needle 5 mm long in order to control that the injection is not too superficial or too deep. All animals were immediately returned to the home cage after the injection procedure. The injected dams were either wild-type or *Shank3* heterozygous. In the following hours, the mice were monitored for sickness behavior, body temperature (measured with a rectal thermometer, Bioseb, France) and abortion. On the consecutive days, weight loss, litter size and pup pre-weaning mortality were also recorded. All pups from a single litter remained with the mother until weaning at postnatal day (PND) 21 to PND28.

### Blood serum collection and Interleukin 6 protein concentration analysis

In addition, blood serum was collected from several pregnant dams for subsequent Interleukin 6 (IL-6) protein concentration analysis. Mice were deeply anesthetized by CO_2_ inhalation, reflexes were checked by a pinch between the toes with tweezers, then the mouse was placed on its back, and the limbs were fixed on the dissection table with duct tape. Next, the mouse belly was sprayed with 70% ethanol and a cardiac puncture was performed using a 1 ml syringe and a 26 G needle. The heart was approached from under the sternum, and negative pressure was gently applied to the syringe plunger. On average 500–700 ml of blood was collected in a 1.5 ml Eppendorf tube. The mouse was then killed by decapitation. Blood was left for 20 min at RT and then centrifuged at 3000 rpm for 10 min at 4 °C. The supernatant was collected, fast-frozen in liquid nitrogen and stored at − 80 °C. Then, the samples were shipped in a package with dry ice to Abcam, USA, where the analysis of the protein concentration of IL-6 in the blood serum of the injected dams was performed using the Fireplex-384 Cytokines (Mouse) Immunoassay Panel (Abcam, Cat. # ab252376).

### Behavioral analyses

After weaning, the male offspring of the injected dams were housed with same-sex littermates in groups of two to four. When they reached five months of age, they were transferred to the behavior room container and left undisturbed (except for weekly cage changes) for two weeks before the start of the experiments for habituation to the new conditions. There they were housed under the following conditions: temperature of 20 ± 1 °C, 30–60% humidity with a 12 h light/dark cycle and provided with food and water ad libitum. Offspring from 3–6 different mothers were used per group. All behavioral experiments were conducted between 9 am and 6 pm during the light phase of the circadian cycle. At least one hour before behavioral testing mice were habituated to the test room. Several behavioral tests were conducted with the same mice, and only one behavioral test was carried out per day. To prevent the mice from being influenced in their behavior by any scent marks of their predecessor, all equipment was cleaned with soap water before and between the individual test procedures [40, 41]. All experimental mice underwent the same sequence of behavioral tests over a three-week period. Order of testing was: (1) Marble burying; (2) Open field; (3) Three-chamber test; (4) Same-sex reciprocal social interaction; (5) Repetitive self-grooming and digging; and (6) Rotarod.

#### Three-chamber test for sociability and preference for social novelty

The test was performed as previously described by [16]. The apparatus consisted of a Plexiglas box (60 cm × 40 cm × 22 cm, L x W x H), which was divided into three separate chambers. (Light intensity in each chamber was 15 lx.) In the front and back chambers, there were cylindrical cages (17 cm high, diameter 8 cm; distance between bars 7 mm, diameter of the bar 3 mm). The individual chambers were connected by sliding doors (7 cm × 8.5 cm, L x H). First, the tested mouse was allowed to explore the whole empty arena freely, with all doors open for 10 min (habituation phase). Then, the mouse was restricted in the central compartment, while an unfamiliar mouse of the same strain (C57BL/6JRj) and sex (stranger 1) was placed inside one of the cylindrical cages and an empty cage was placed in the opposite chamber. The tested mouse was then allowed to explore the whole apparatus for 10 min (sociability phase). Finally, another unfamiliar mouse of the same strain (C57BL/6JRj) and sex (stranger 2) was placed inside the previously empty cage. The tested mouse could then again freely explore the whole apparatus for 10 min (social novelty preference phase). In all three phases, time spent in each compartment was automatically recorded with the video tracking software Viewer3 from Biobserve. The arena and the cages were cleaned with soap water between subjects.

#### Same-sex reciprocal social interaction (RSI)

The test was performed as previously described by [42] with few modifications. In order to increase the social motivation of the male mice, they were housed individually for a period of 4 weeks [43]. The tested mouse was left 20 min for habituation in a clean standard cage (Plexiglas, 36 cm × 20 cm × 14 cm, L x W x H; 24 lx) filled with fresh litter. After this time, an unfamiliar mouse of the same strain (C57BL/6JRj) and sex and of similar age and weight was introduced. The two animals were allowed to interact freely for five minutes. Behavior was recorded in a sound-proof and anechoic room. Social interactions were videotaped, and the pre-recorded video was then analyzed with the Ethovision XT 15 software from Noldus. Ultrasonic vocalizations (USVs) were recorded with a condenser ultrasound microphone CM16/CMPA placed 33 cm above the cage, the interface UltraSoundGate 116H and the software Avisoft SASLab Pro Recorder from Avisoft Bioacoustics (sampling frequency: 300 kHz; fast Fourier transform length: 1024 points; 16-bit format). The sound recording was analyzed with VocalMat [44], a MATLAB-based software. The time spent in contact (s), the number of contacts and the total number of calls were analyzed. The distribution of the different USV types was also assessed. First, because of the considerable inter-individual variability in the number of calls emitted, the types of vocalizers were determined by a quartile analysis. All data were arranged by increasing order and then divided into four parts, each representing 25%. The first quartile (Q1) presented the value below which the mice were categorized as “low” vocalizers. The second quartile (Q2) represented the median, and the third quartile (Q3) presented the value above which the mice were categorized as “high” vocalizers. “Average” vocalizers were all the mice found between Q1 and Q3. Only the “average” and “high” vocalizers were used in the USV types analysis.

#### Repetitive self-grooming and digging

The tested mouse was placed in a standard cage (36 cm × 20 cm × 14 cm) filled with fresh litter, where it could explore freely for 20 min. The light intensity was 24 lx. The first 10 min of habituation was not scored. During the second 10 min, the cumulative time spent self-grooming (s) as well as the total number of grooming episodes were manually measured by a blinded observer. The self-grooming was assessed during the habituation phase of the RSI test. The time spent grooming was divided by the total number of grooming episodes in order to attest the grooming bout duration (s). In addition, the total time spent digging (s) during the same test was also manually scored.

#### Open field

The tested mouse was placed in a corner of a large empty arena (50 cm × 50 cm × 40 cm, L x W x H) and allowed to explore freely for a period of 30 min. The light intensity in the center of the arena was 10 lx. The total distance traveled (cm), the time spent in the center zone (s) and the number of entries into the center zone were determined by the video tracking software Viewer 3 (Biobserve).

#### Marble burying

The test was performed as previously described with few modifications [45]. A standard cage (36 cm × 20 cm × 14 cm, L x W x H) was filled 5 cm high with fresh litter, which was slightly pressed to the ground to achieve a flat, even surface. Then, 18 marbles (diameter: 1.4 mm) evenly spaced were positioned in the cage. The light intensity was 100 lx. A mouse was placed in the corner of the cage and allowed to explore freely. After 30 min, the mouse was put back into its home cage and the number of buried marbles (2/3 depth) was noted.

#### Rotarod

The Rotarod test was used to determine the motor coordination and motor learning of the mice. The apparatus consisted of a 3-cm-thick rotating cylinder. Before each trial, the cylinder was first moved to the hold position with the lowest speed of 4 rpm and the mice were placed on the cylinder for 30 s. Then, the speed was gradually accelerated from 4 to 40 rpm within five minutes. The attempt was over when the mouse fell from the cylinder or clung passively onto the cylinder. Three trials per mouse were carried out, with five minutes breaks between them, during which the mouse was put back into its home cage (food and water ad libitum). The same was repeated for three consecutive days. After each trial, the end speed (rpm) was noted.

### Subcellular fractionation of protein lysate and Western Blot procedure

Mice were deeply anesthetized by CO_2_ inhalation, reflexes were checked by a pinch between the toes with tweezers, and then, the mice were killed by decapitation. The adult brains were extracted, fast-frozen in liquid nitrogen and then stored at − 80 °C until dissection. From both hemispheres, prefrontal cortexes (PFC), the complete striata and hippocampi were dissected under a light microscope. The dissection of the PFC was based on two publications: [46, 47]. A subcellular fractionation was performed as published previously [48]. Tissues were homogenized in buffer 1 containing 10 mM HEPES pH 7.4, 2 mM EDTA, 5 mM sodium orthovanadate, 30 mM sodium fluoride, 20 mM β-glycerolphosphate, protease inhibitor cocktail (Roche). Samples were centrifuged at 500 × g for 5 min at 4 °C. Resulting supernatants were centrifuged at 10,000 × g for 15 min at 4 °C. After the centrifugation, pellets were resuspended in buffer 2 composed of 50 mM HEPES pH 7.4, 2 mM EDTA, 2 mM EGTA, 5 mM sodium orthovanadate, 30 mM sodium fluoride, 20 mM β-glycerolphosphate, 1% Triton-X-100, protease inhibitor cocktail (Roche) and centrifuged at 20,000 × g for 80 min at 4 °C. Finally, pellets were resuspended in buffer 3 containing 50 mM Tris pH 9, 5 mM sodium orthovanadate, 30 mM sodium fluoride, 20 mM β-glycerolphosphate, 1% NaDOC, protease inhibitor cocktail (Roche), snap-frozen in liquid nitrogen and then stored at − 80 °C. The success of the subcellular fractionation was confirmed via Western Blot analysis for PSD95 and SHANK3 (postsynaptic) and SYNAPTOPHYSIN (SYN, presynaptic). The total protein concentration of the samples was determined by a Bradford assay. Equal amounts (3 μg for striatum and hippocampus; 2 μg for PFC) of each sample were separated using SDS–PAGE and subsequently blotted on nitrocellulose membranes according to standard protocols. The protein ladder Spectra Broad Range (Thermo Scientific, cat. 26634) was used in most of the experiments, except for the determination of the subcellular fractionation of the striatum of offspring of Het dams, where Spectra High Range was used (Thermo Scientific, cat. 26625). After the transfer of the proteins, the membrane was cut in three parts at 140 kDa and at 70 kDa. The upper part was then incubated with antibodies detecting proteins of high molecular weight, the middle part—proteins of middle molecular weight and the lower part—with proteins of small molecular weight. In addition, in several experiments, the lower membrane was once incubated over night with HOMER and after development and three washing steps, it was once again incubated over night with ß-ACTIN. Incubation with a primary antibody (β-ACTIN, ms, Sigma, cat. A5316; SHANK2, rb, Homemade [16]; SHANK3, rb, Homemade Frag 1 + 2, Tier 2 [16]; mGluR5, rb, Millipore, cat. AB5675; PSD95, ms, Abcam, cat. ab2723; HOMER1b/c, rb, Synaptic Systems, cat. 160 022, GAPDH, ms, Thermo Fisher, cat MA5 15,738-D800) was followed by treatment of the membrane with HRP-conjugated secondary antibodies (goat anti-rabbit, 1:1000 and rabbit anti-mouse, 1:3000; all Dako, Hamburg, Germany). The signals were finally visualized with ECL Western Blotting substrate (Pierce) and the MicroChemi 4.2 machine. All signals were quantified using Gel analyzer software (www.gelanalyzer.com/) and normalized first against the values of the respective signal for β-ACTIN and second against the mean value of all bands loaded into the same gel.

### Data analysis

All of the behavioral and biochemical experiments and all data analyses were performed under blinded conditions in which the persons performing the experiment and the persons performing the analysis used a random numerical code, which was produced and known by another person, to label the different samples.

### Statistical analysis

Data are shown as mean ± SEM. Significances are stated with *p* values. Significance level was set to 0.05 (*p < 0.05, **p < 0.01, ***p < 0.001, ****p < 0.0001) with a corresponding 95% confidence interval, and tendencies were also displayed (#*p* < 0.10). The biological replicates from three mice were used for the biochemical experiments with two technical replicates. For the behavioral experiments, 6–12 offspring of 3–6 mothers were used. All data were tested for normality with the Shapiro–Wilk test. For the analysis of Interleukin 6 blood serum concentration in the injected dams and for the Western Blot results with tissue from the offspring of *Shank3* heterozygous mothers, a two-way ANOVA followed by a Bonferroni correction for multiple comparisons was used. In other experiments, when only two groups were compared, data were analyzed by an unpaired two-tailed t test (when normal) or by Mann–Whitney two-tailed test (when not normal). When three groups were compared, data were analyzed by one-way ANOVA followed by a Bonferroni correction for multiple comparisons (when normal) and by Kruskal–Wallis followed by Dunn’s correction for multiple comparisons (when not normal). Two-sided Fisher’s exact test (expected frequencies < 5) or Pearson’s chi-square test (expected frequencies ≥ 5) was used in the analysis of categorical data when comparing two to three groups. Pairwise Fisher’s exact test followed by a Benjamini–Hochberg correction for multiple comparisons was used in the analysis of categorical data when comparing eight groups. In the behavioral experiments, a two-way ANCOVA followed by a Bonferroni correction for multiple comparisons was used with a few exceptions. The Rotarod test was analyzed by a linear mixed model analysis with repeated measures followed by a Tukey–Kramer correction for multiple comparisons. The three-chamber test was analyzed by a paired two-tailed t test (when normal) or by a Wilcoxon matched-pairs signed rank two-tailed test (when not normal) comparing the time spent in two chambers within each group. The two-way ANCOVA, Pearson chi-square test, Fisher’s exact test and the quartile analysis of the mouse ultrasonic vocalizations (USVs) were performed with SPSS, version 27 (IBM) by Ekaterina Atanasova. Pairwise Fisher’s exact test followed by a Benjamini–Hochberg correction for multiple comparisons was performed with R software by Valentin Ioannidis. The linear mixed model analysis with repeated measures followed by a Tukey–Kramer correction for multiple comparisons was performed with SAS software (SAS Institute) by Prof. Dr. Benjamin Mayer. All the other statistical tests were performed with GraphPad Prism 8 by Ekaterina Atanasova.

## Results

### Two-hit ASD model

**Fig. 1:**
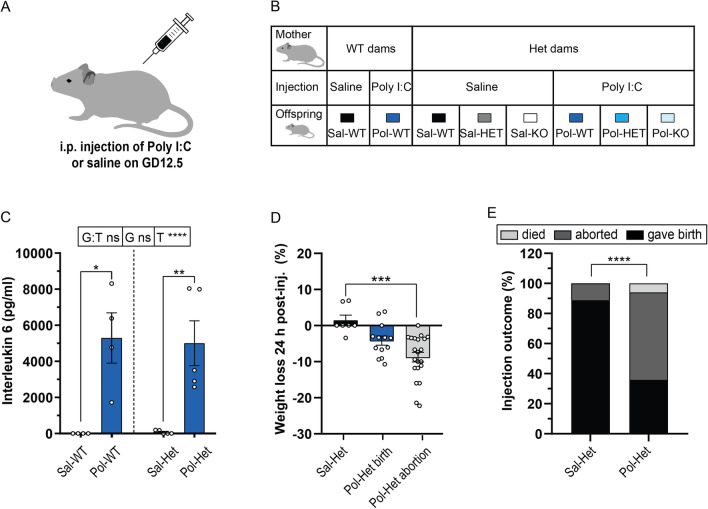
Experimental design and dam response to the injection of the synthetic virus. **A** An intraperitoneal (i.p.) injection of saline or Poly I:C was performed in pregnant mice on gestational day (GD) 12.5. **B** Table describing all the experimental groups. **C** Interleukin 6 blood serum concentration (pg/ml) three hours post-injection. T ****p < 0.0001, Sal-WT vs Pol-WT *p = 0.0114 and Sal-Het vs Pol-Het **p = 0.0085. Data were analyzed by two-way ANOVA followed by a Bonferroni correction for multiple comparisons. Sal-WT *n* = 4, Pol-WT *n* = 4, Sal-Het *n* = 5, Pol-Het *n* = 5. G = Genotype of the mother; T = Treatment of the mother; and G:T = interaction between the two factors. **D** Weight lost (%) 24 h post-injection. Sal-Het vs Pol-Het abortion ***p = 0.0005. Data were analyzed by Kruskal–Wallis followed by Dunn’s correction for multiple comparisons. Sal-Het *n* = 7, Pol-Het birth *n* = 13, Pol-Het abortion *n* = 21. **E** Injection outcome (%). Sal-Het vs Pol-Het ****p < 0.0005. Data were analyzed by Pearson chi-square test (expected frequencies ≥ 5), two-sided. Sal-Het birth *n* = 16, Sal-Het abortion *n* = 2, Pol-Het birth *n* = 24, Pol-Het abortion *n* = 39, Pol-Het death *n* = 4. **C**–**E** Data were tested for normality with the Shapiro–Wilk test. Significance level was set to 0.05 (# < 0.10, *p < 0.05, **p < 0.01, ***p < 0.001, ****p < 0.0001). Mean ± SEM. *ns* not significant

Wild-type and *Shank3* heterozygous pregnant mice received an intraperitoneal injection of the synthetic virus Poly I:C or saline on GD12.5 (Fig. 1A). Heterozygous breedings lead to the creation of six experimental groups—wild-type (WT), *Shank3* heterozygous (Het) and knockout (KO) offspring of a saline-injected and poly I:C-injected *Shank3* Het dams, respectively (Fig. 1B). To control for a possible impact of the genotype of the dams, wild-type dams were injected as well (Fig. 1B). The occurrence of a systemic inflammation in those mice was confirmed by an ELISA analysis for Interleukin 6 (IL-6). Three hours post-injection, there was a significant increase in the IL-6 concentration in the blood serum of both WT and *Shank3* Het mice (Fig. 1C). The genotype of the mother had no effect indicating that both dams reacted similarly to the injection of the synthetic virus. The inflammation was transitory, since nine hours post-injection, no significant differences in IL-6 concentration were found (Additional file 1: Fig S1A). Moreover, the treatment with Poly I:C led to a significant decrease in body temperature three to nine hours post-injection (Additional file 1: Fig S1B and C). The temperature was back to normal 24 h after the injection of Poly I:C; however, the dams that suffered an abortion had a significant weight loss (Fig. 1D and Additional file 1: Fig S1D). This weight loss was indicative of the abortion as in the Poly I:C group 55–60% of the mice lost the embryos (Fig. 1E and Additional file 1: Fig S1E). The Poly I:C treatment did not lead to premature birth (Additional file 1: Fig S1F and G) but showed an increase in the pre-weaning mortality (Additional file 1: Fig S1H and I). Both groups (saline and Poly I:C) gave birth to a similar number of pups (Additional file 1: Fig S1J and K) presenting expected sex and genotype ratios (Additional file 1: Fig S1L-O) implying that two-hit mice were not less likely to survive. Finally, the weight gain from conception until the day before the injection was comparable between all groups meaning that the higher abortion rate in the poly I:C-injected dams was not due to them carrying more embryos (Additional file 1: Fig S1P and Q). The Poly I:C injection caused a severe reaction in the dams manifesting with a transitory systemic inflammation and body temperature decrease that was leading to higher abortion rates and increased pre-weaning mortality of the pups.

### Core symptoms of autism

**Fig. 2:**
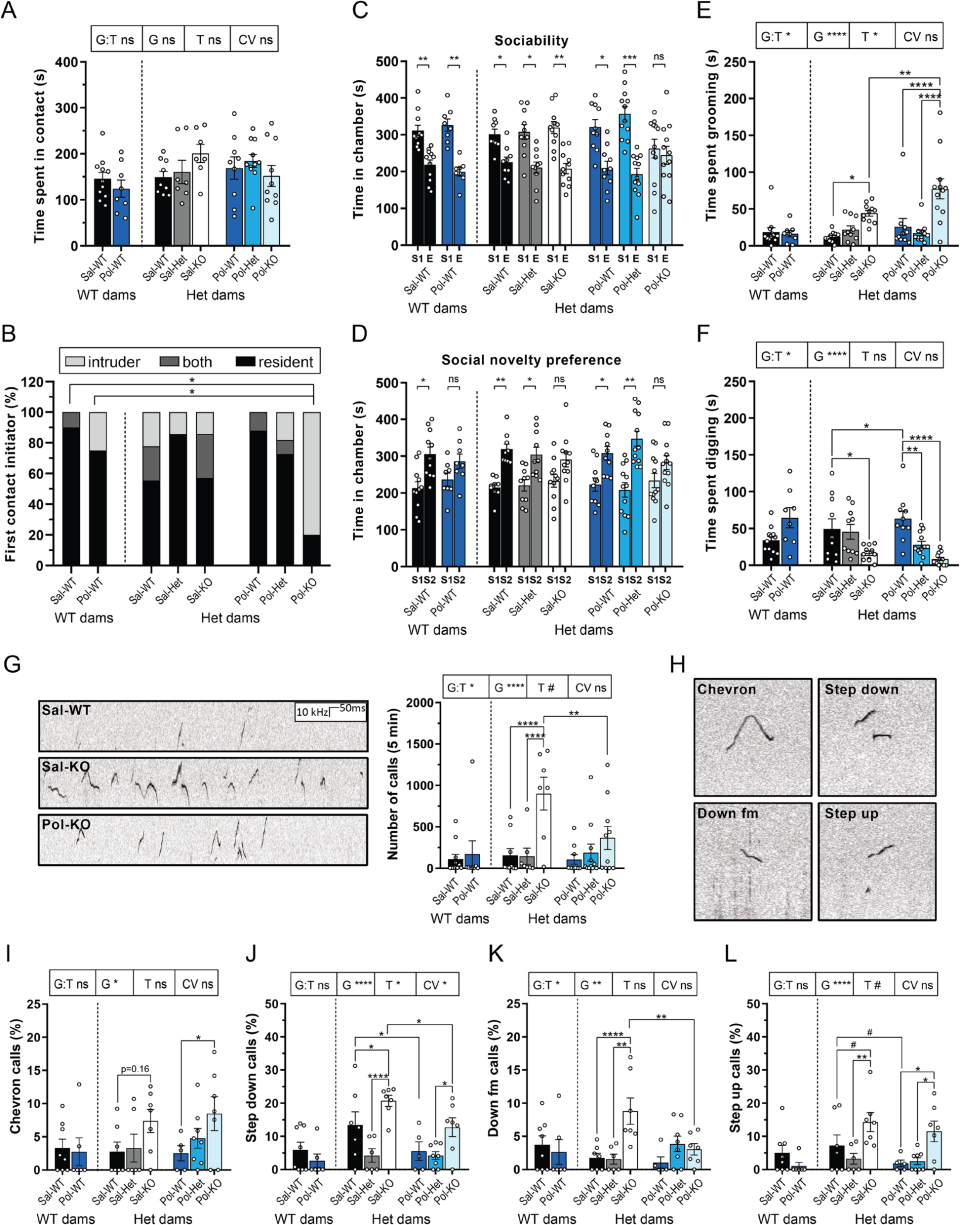
Core symptoms of autism. **A** Time spent in contact (s) during same-sex reciprocal social interaction. Data were analyzed by two-way ANCOVA followed by a Bonferroni correction for multiple comparisons. *G* Genotype of the offspring; *T* Treatment of the mother; *G:T* interaction between the two factors; and *CV* covariate “Genotype of the mother.” **B** Initiator of the first contact (%) during same-sex reciprocal social interaction. Pol-KO vs the following: Sal-WT (WT dams) **p = 0.0052 and Pol-WT (WT dams) **p = 0.0018. Data were analyzed by pairwise Fisher’s exact test followed by a Benjamini–Hochberg correction for multiple comparisons. **C** Time spent in chamber (s) during the sociability phase of the three-chamber test. Sal-WT (WT dams) **p = 0.0061, Pol-WT (WT dams) **p = 0.0025, Sal-WT (Het dams) *p = 0.0199, Sal-Het *p = 0.0215, Sal-KO **p = 0.0037, Pol-WT (Het dams) *p = 0.0138, Pol-Het ***p = 0.0007. **D** Time spent in chamber (s) during the social novelty preference phase of the three-chamber test. Sal-WT (WT dams) *p = 0.0285, Sal-WT (Het dams) **p = 0.0019, Sal-Het *p = 0.0327, Pol-WT (Het dams) *p = 0.0389, Pol-Het **p = 0.0048. **E** Time spent self-grooming (s). G:T *p = 0.0310, G ****p < 0.0005 and T *p = 0.0450, Sal-KO vs Sal-WT *p = 0.0210 and Pol-KO vs the following: Sal-KO **p = 0.0020, Pol-WT ****p < 0.0005 and Pol-Het ****p < 0.0005. **F** Time spent digging (s). G:T *p = 0.0110 and G ****p < 0.0005, Sal-WT vs the following: Pol-WT *p = 0.0100 and Sal-KO *p = 0.0260. Pol-WT vs the following: Pol-Het **p = 0.0010 and Pol-KO ****p < 0.0005. **G** Spectrogram of calls emitted by Sal-WT, Sal-KO and Pol-KO mice. Total number of calls emitted over 5 min during same-sex reciprocal social interaction. G:T *p = 0.0190, G ****p < 0.0005, T #*p* = 0.0660, Sal-KO vs the following: Sal-WT ****p < 0.0005, Sal-Het ****p < 0.0005 and Pol-KO **p = 0.002. Data were analyzed by two-way ANCOVA followed by a Bonferroni correction for multiple comparisons. Sal-WT (WT dams) *n* = 11, Pol-WT (WT dams) *n* = 8, Sal-WT (Het dams) *n* = 9, Sal-Het *n* = 7, Sal-KO *n* = 7, Pol-WT (Het dams) *n* = 9, Pol-Het *n* = 11, Pol-KO *n* = 10. **H** Spectrograms of chevron, step down, down fm and step up call types. fm = frequency modulation. **I** Percentage of chevron calls. G *p = 0.0130. Sal-WT vs Sal-KO *p* = 0.1660 and Pol-WT vs Pol-KO *p = 0.0480. **J** Percentage of step down calls. G ****p < 0.0005, T *p = 0.0190, CV *p = 0.0330, Sal-KO vs the following: Sal-WT *p = 0.0390, Sal-Het ****p < 0.0005 and Pol-KO *p = 0.0240. Sal-WT vs Pol-WT *p = 0.0400. Pol-Het vs Pol-KO *p = 0.0400. **K** Percentage of down fm calls. G:T *p = 0.0100, G **p = 0.0030, Sal-KO vs the following: Sal-WT ****p < 0.0005, Sal-Het **p = 0.0010 and Pol-KO **p = 0.0020. **L** Percentage of step up calls. G ****p < 0.0005, T *p* = 0.1190, Sal-KO vs the following: Sal-WT #*p* = 0.0510 and Sal-Het **p = 0.0050. Pol-KO vs the following: Pol-WT *p = 0.0160 and Pol-Het *p = 0.0170. Sal-WT vs Pol-WT #*p* = 0.0590. **A**–**B** Sal-WT (WT dams) *n* = 11, Pol-WT (WT dams) *n* = 8, Sal-WT (Het dams) *n* = 9, Sal-Het *n* = 7, Sal-KO *n* = 7, Pol-WT (Het dams) *n* = 9, Pol-Het *n* = 11, Pol-KO *n* = 10. **C**–**D** Data were analyzed by a paired two-tailed t-test (when normal) or by Wilcoxon matched-pairs signed rank two-tailed test (when not normal). Sal-WT (WT dams) *n* = 11, Pol-WT (WT dams) *n* = 8, Sal-WT (Het dams) *n* = 9, Sal-Het *n* = 10, Sal-KO *n* = 11, Pol-WT (Het dams) *n* = 10, Pol-Het *n* = 12, Pol-KO *n* = 12. E = Empty cage, S1 = Stranger 1, S2 = Stranger 2. **E**–**F** Data were analyzed by two-way ANCOVA followed by a Bonferroni correction for multiple comparisons. Sal-WT (WT dams) *n* = 12, Pol-WT (WT dams) *n* = 8, Sal-WT (Het dams) *n* = 10, Sal-Het *n* = 10, Sal-KO *n* = 11, Pol-WT (Het dams) *n* = 10, Pol-Het *n* = 12, Pol-KO *n* = 12. G = Genotype of the offspring; T = Treatment of the mother; G:T = interaction between the two factors; and CV = covariate “Genotype of the mother.” **I-L** Data were analyzed by two-way ANCOVA followed by a Bonferroni correction for multiple comparisons. Sal-WT (WT dams) *n* = 11, Pol-WT (WT dams) *n* = 8, Sal-WT (Het dams) *n* = 9, Sal-Het *n* = 7, Sal-KO *n* = 7, Pol-WT (Het dams) *n* = 9, Pol-Het *n* = 11, Pol-KO *n* = 10. **A**–**L** Data were tested for normality with the Shapiro–Wilk test. Significance level was set to 0.05 (# < 0.10, *p < 0.05, **p < 0.01, ***p < 0.001, ****p < 0.0001). Mean ± SEM. *ns* not significant

Next, the core symptoms of ASD were assessed in the adult offspring of the injected dams. First, alterations in social behavior and communication were investigated. During the same-sex reciprocal social interaction test, male mice spent the same amount of time in contact (Fig. 2A) and made the same number of contacts (Additional file 1: Fig S2A). Mice that displayed excessive aggression were excluded from the analysis as the goal of the test was to assess exclusively affiliative social interaction. Moreover, the two-hit mice were not the ones to initiate the first contact (Fig. 2B). This social approach deficit was confirmed during phase two of the three-chamber test where the two-hit mice were the only group that did not show a preference in spending time with their conspecific (Fig. 2C). During the last phase of the test, Pol-WT, Sal-KO and two-hit mice showed a social novelty recognition alteration (Fig. 2D). Furthermore, during the habituation phase of the three-chamber test all groups showed no innate preference toward one of the rooms (Fig S2B).

Addressing the second core symptom of ASD—repetitive behavior—the two-hit mice spent a significantly longer time self-grooming compared to the Pol-WT and Sal-KO groups (Fig. 2E). In addition, each grooming event of the mice in the two KO groups lasted longer resulting in an increase in the grooming bout duration (Additional file 1: Fig S2C). Contrary to the Sal-KO group, the Pol-KO mice did not spend more time self-grooming (Fig. 2E). However, the Pol-WT mice displayed a significant increase in the time spent digging, while the KO groups showed a decrease in this behavior (Fig. 2F).

In addition, the Sal-KO mice emitted a significantly higher amount of ultrasonic vocalizations (Fig. 2G). Two-hit mice presented a decrease in the number of calls compared to Sal-KO (Fig. 2G). Moreover, Sal-KO male mice were the only group that did not contain any “low” vocalizers (Additional file 1: Fig S3A). Therefore, in the qualitative analysis of the different call types emitted by the mice, only the “average” and “high” vocalizers were included, while all mice that emitted less than 5.25 calls in total were excluded (Additional file 1: Fig S3B). After classifying the calls, chevron, step down, down fm (frequency modulation) and step up calls seemed to be altered in Sal-KO and Pol-KO (Fig. 2H). Both KO groups emitted more chevron (Fig. 2I) and step down calls (Fig. 2J) yet only Sal-KO mice presented with a higher amount of down fm calls (Fig. 2K), all three being USV types ending at a lower frequency. Moreover, Pol-WT mice emitted less step down (Fig. 2J) and step up calls (Fig. 2L), indicating that the Poly I:C treatment caused a decrease in the number of calls with frequency jumps in the WT offspring. Finally, both KO mice emitted more step up calls (Fig. 2L). No further differences in USV types were found between the groups except for a decrease in the percentage of short calls emitted by Sal-KO mice compared to Sal-Het (Additional file 1: Fig S4).

### Autism-related comorbidities

**Fig. 3:**
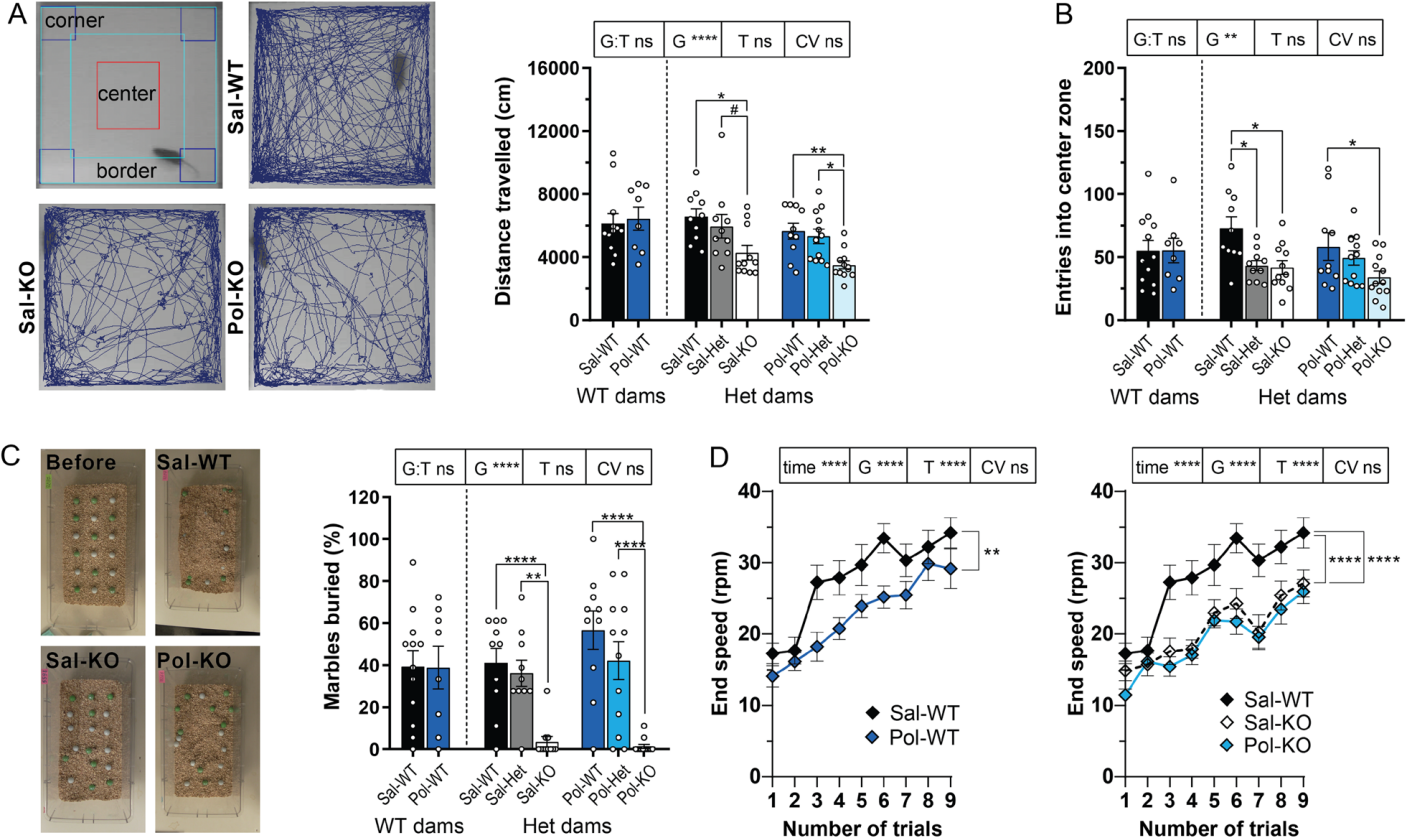
Comorbidities often associated with ASD. **A** Representative figures of the zones in the open field arena and the distance travelled by Sal-WT, Sal-KO and Pol-KO. Total distance travelled (cm) in the open field arena. G ****p < 0.0005, Sal-KO vs the following: Sal-WT *p = 0.0200 and Sal-Het #*p* = 0.0950. Pol-KO vs the following: Pol-WT **p = 0.0020 and Pol-Het *p = 0.0400. **B** Number of entries into the center zone of the open field. G **p = 0.0010, Sal-WT vs the following: Sal-Het *p = 0.0350 and Sal-KO *p = 0.0190. Pol-WT vs Pol-KO *p = 0.0140. **C** Representative pictures of the marble burying test—before and after for Sal-WT, Sal-KO and Pol-KO. Percentage of marbles buried. G ****p < 0.0005, Sal-KO vs the following: Sal-Het **p = 0.0050 and Sal-WT ****p < 0.0005. Pol-KO vs the following: Pol-Het ****p < 0.0005 and Pol-WT ****p < 0.0005. **D** End speed (rpm) during the Rotarod test. G:T ns, time ****p < 0.0001, G ****p < 0.0001 and T ****p < 0.0001, Sal-WT vs Pol-WT **p = 0.0049. Sal-WT vs the following: Sal-KO ****p < 0.0001 and Pol-KO ****p < 0.0001. Sal-WT (WT dams) *n* = 12, Pol-WT (WT dams) *n* = 8, Sal-WT (Het dams) *n* = 10, Sal-Het *n* = 8, Sal-KO *n* = 11, Pol-WT (Het dams) *n* = 10, Pol-Het *n* = 6, Pol-KO *n* = 12. **A**–**C** Sal-WT (WT dams) *n* = 12, Pol-WT (WT dams) *n* = 8, Sal-WT (Het dams) *n* = 10, Sal-Het *n* = 10, Sal-KO *n* = 11, Pol-WT (Het dams) *n* = 10, Pol-Het *n* = 12, Pol-KO *n* = 12. **A**–**D** Data were tested for normality with the Shapiro–Wilk test. Rotarod data were analyzed by a linear mixed model analysis with repeated measures followed by a Tukey–Kramer correction for multiple comparisons and all other data by two-way ANCOVA followed by a Bonferroni correction for multiple comparisons. Significance level was set to 0.05 (# < 0.10, *p < 0.05, **p < 0.01, ***p < 0.001, ****p < 0.0001). Mean ± SEM. *G* Genotype of the offspring; *T* Treatment of the mother; *G:T* interaction between the two factors; *CV* covariate “Genotype of the mother”; and *ns* not significant

Both KO groups showed a similar decrease in locomotion (Fig. 3A). Those mice spent the same amount of time in the center of the open field arena as the other groups (Additional file 1: Fig S5A), but they made fewer entries into the center (Fig. 3B). Moreover, Sal-KO and Pol-KO male mice showed an avoidance behavior during the marble burying test where they would not bury the objects (Fig. 3C). Finally, the motor abilities of the adult offspring were investigated via the Rotarod test. A motor coordination deficit was present in the WT offspring of poly I:C-treated dams (Fig. 3D). A severe deficit was found in the Sal-KO and two-hit mice, while both KO groups performed in a comparable way during the experiment (Fig. 3D). However, all experimental groups were able to improve their motor coordination over time, meaning that they had no motor learning alterations. The other experimental groups showed no alterations in motor coordination and are displayed in Additional file 1: Fig S5B-E.

### Brain region-specific alterations of postsynaptic proteins in two-hit mice

To comprehend if these behavioral changes co-occur with biochemical alterations, brain tissue lysates of the eight offspring groups have been collected (see Fig. 1B). Homogenates and the P3 fraction of striatum, hippocampus and prefrontal cortex (PFC) have been analyzed using Western Blotting, to compare protein expression in total protein lysate and the postsynaptic density. The SHANK3 antibody used in this study was validated in a recent paper [49]. With the loading control used in this analysis (ß-ACTIN), a test was performed where the ß-ACTIN expression was normalized over another possible loading control GAPDH (Additional file 1: Fig S6). As no differences were found, we concluded that ß-ACTIN is a suitable loading control for the brain tissue Western Blot analysis. Successful fractionation of the brain tissue of the offspring of WT dams was confirmed by Western Blotting for PSD95 (postsynaptic) and SYNAPTOPHYSIN (SYN, presynaptic) (Additional file 1: Fig S7). Poly I:C injection in WT dams only revealed minor changes in the tissue homogenate of the WT offspring, but no changes in the P3 fractions (Additional file 1: Fig S8-13). In striatum, only the SHANK3c/d isoform was slightly increased in homogenate (Additional file 1: Fig S8A). In hippocampus, the SHANK3e isoform was slightly increased, and HOMER1b/c slightly decreased in homogenate (Additional file 1: Fig S10A and S10G) and no changes were observed in PFC (Additional file 1: Fig S12-13).

**Fig. 4:**
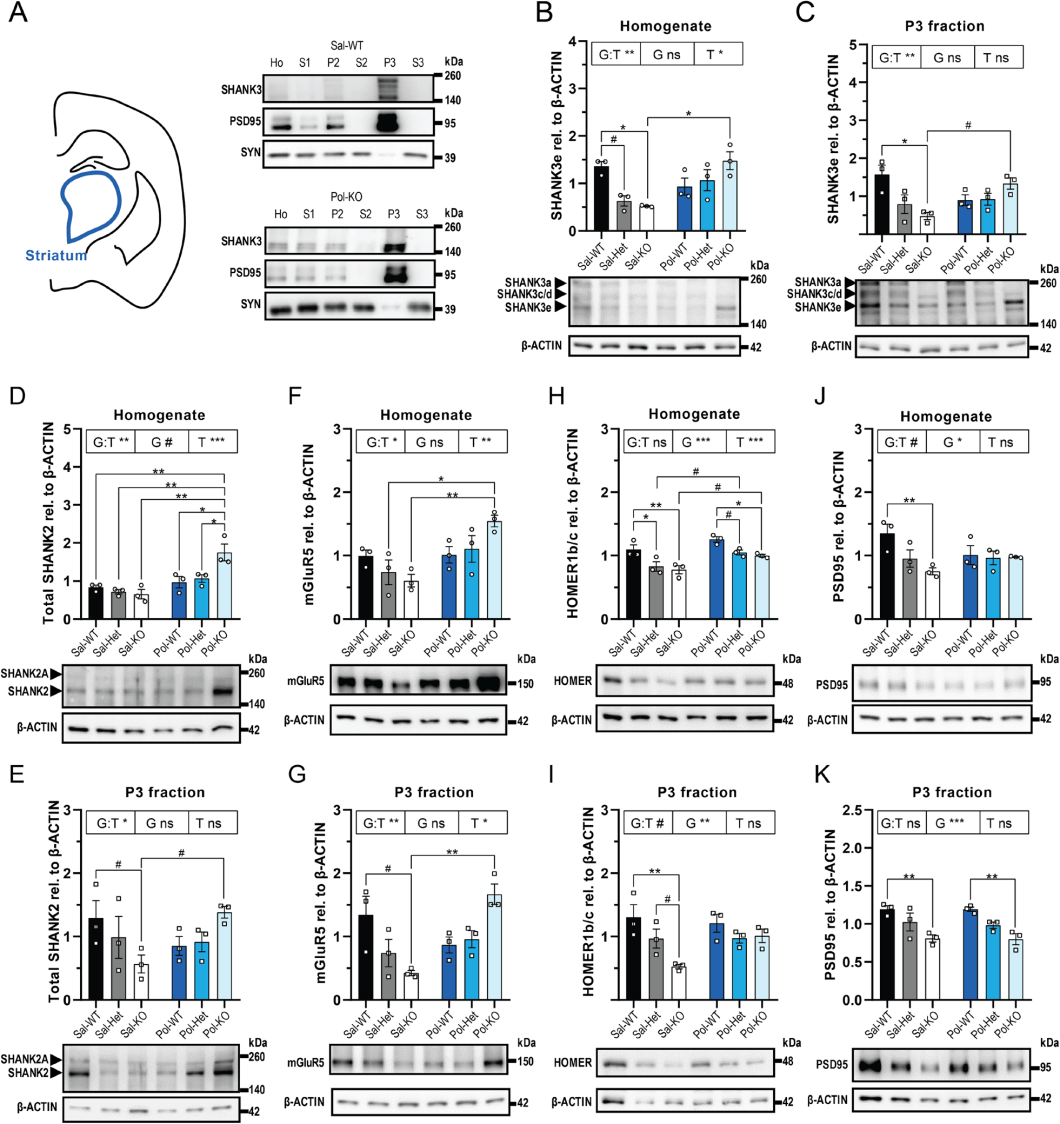
Synaptic changes in striatum of the offspring of Het dams. **A** Fractionation of the striatum. Western Blots of Sal-WT and Pol-KO are shown for SHANK3, PSD95 (postsynaptic) and SYNAPTOPHYSIN (SYN, presynaptic)**. B** Western Blot analysis for SHANK3 and β-ACTIN of striatum homogenate. The SHANK3e isoform was analyzed. G:T **p = 0.0021; T *p = 0.0227, Pol-KO vs Sal-KO *p = 0.0114, Sal-WT vs Sal-KO *p = 0.0301, Sal-WT vs Sal-Het #*p* = 0.0756. **C** Western Blot analysis for SHANK3 and β-ACTIN of striatum P3 fraction. The SHANK3e isoform was analyzed. G:T **p = 0.0040, Pol-KO vs Sal-KO #*p* = 0.0842, Sal-KO vs Sal-WT *p = 0.0155. **D** Western Blot analysis for SHANK2 and β-ACTIN of striatum homogenate. The total SHANK2 was analyzed. G:T **p = 0.0075, G #*p* = 0.0585, T ***p = 0.0004, Pol-KO vs the following: Sal-WT **p = 0.0052, Sal-Het **p = 0.0018, Sal-KO **p = 0.0010, Pol-WT *p = 0.0173, Pol-Het *p = 0.0440. **E** Western Blot analysis for SHANK2 and β-ACTIN of striatum P3 fraction. The total SHANK2 was analyzed. G:T *p = 0.0296, Sal-KO vs Pol-KO #*p* = 0.0510, Sal-WT vs Sal-KO #*p* = 0.0909. **F** Western Blot analysis for mGluR5 and β-ACTIN of striatum homogenate. G:T *p = 0.0236, T **p = 0.0028, Sal-Het vs Pol-KO *p = 0.0285, Sal-KO vs Pol-KO **p = 0.0091. **G** Western Blot analysis for mGluR5 and β-ACTIN of striatum P3 fraction. G:T **p = 0.0017, T *p = 0.0450, Sal-KO vs Pol-KO **p = 0.0058, Sal-WT vs Sal-KO #*p* = 0.0565. **H** Western Blot analysis for HOMER1b/c and β-ACTIN of striatum homogenate. G ***p = 0.0006, T ***p = 0.0010, Sal-WT vs Sal-KO **p = 0.0055, Pol-WT vs Pol-KO *p = 0.0221, Sal-WT vs Sal-Het *p = 0.0184, Pol-WT vs Pol-Het #*p* = 0.0702, Pol-Het vs Sal-Het #*p* = 0.0542, Pol-KO vs Sal-KO #*p* = 0.0505. **I** Western Blot analysis for HOMER1b/c and β-ACTIN of striatum P3 fraction. G:T #*p* = 0.0928, G **p = 0.0085, Sal-WT vs Sal-KO **p = 0.0032, Sal-Het vs Sal-KO #*p* = 0.0954. **J** Western Blot analysis for PSD95 and β-ACTIN of striatum homogenate. G:T #*p* = 0.0761, G *p = 0.0447, Sal-WT vs Sal-KO **p = 0.0084. **K** Western Blot analysis for PSD95 and β-ACTIN of striatum P3 fraction. G ***p = 0.0003, Sal-WT vs Sal-KO **p = 0.0042, Pol-WT vs Pol-KO **p = 0.0035. **B**–**K** Data were tested for normality with the Shapiro–Wilk test followed by two-way ANOVA with a Bonferroni correction for multiple comparisons. Significance level was set to 0.05 (# < 0.10, *p < 0.05, **p < 0.01, ***p < 0.001, ****p < 0.0001). Mean ± SEM, *n* = 3. *G* Genotype of the offspring; *T* Treatment of the mother; *G:T* interaction between the two factors; and *ns* not significant

However, the offspring of the *Shank3* Het dams were affected by the Poly I:C dependent on their genotype. In striatum, fractionation was conducted successfully (Fig. 4A, Additional file 1: Fig S14A and Fig S15A-C). SHANK3 levels, total as well as individual isoforms, were decreased in the Sal-Het and Sal-KO as expected both in homogenate (Additional file 1: Fig S16A) and the P3 fraction (Additional file 1: Fig S16B). However, SHANK3e was specifically increased in the Pol-KO, both in homogenate (Fig. 4B) and the P3 fraction (Fig. 4C). Moreover, also SHANK2 was significantly increased in the Pol-KO both in homogenate (Fig. 4D) and the P3 fraction (Fig. 4E) and the same was also observed for mGluR5 (Fig. 4F, G). HOMER1b/c and PSD95 were both decreased in the Sal-KO compared to Sal-WT, but Poly I:C had no effect (Fig. 4H, K).

**Fig. 5:**
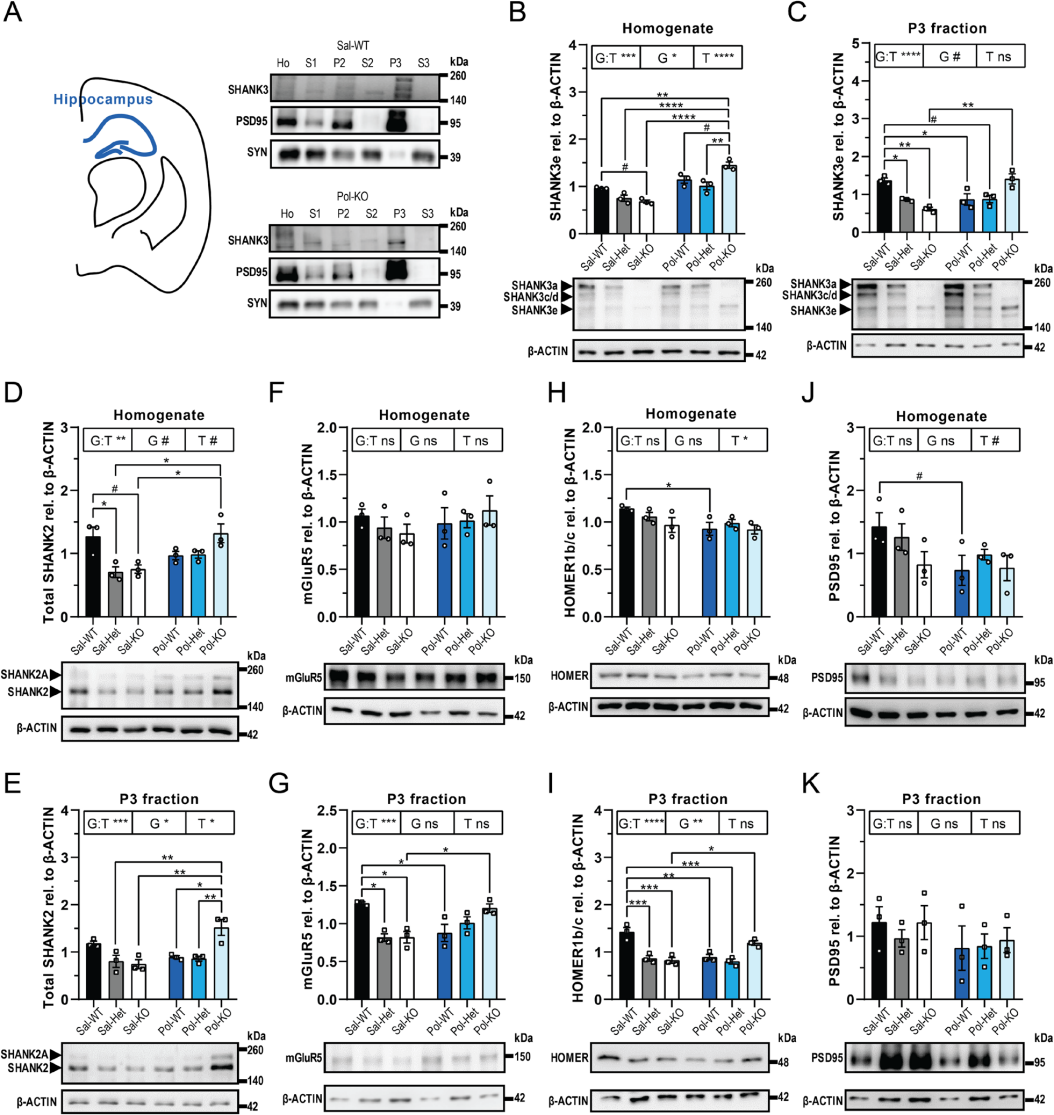
Synaptic changes in hippocampus of the offspring of Het dams. **A** Fractionation of the hippocampus. Western Blots of Sal-WT and Pol-KO are shown for SHANK3, PSD95 (postsynaptic) and SYNAPTOPHYSIN (SYN, presynaptic). **B** Western Blot analysis for SHANK3 and β-ACTIN of hippocampus homogenate. The SHANK3e isoform was analyzed. G:T ***p = 0.0007, G *p = 0.0166, T ****p < 0.0001, Sal-WT vs Pol-KO **p = 0.0017, Sal-Het vs Pol-KO ****p < 0.0001, Sal-KO vs Pol-KO ****p < 0.0001, Pol-WT vs Pol-KO #*p* = 0.0591, Pol-Het vs Pol-KO **p = 0.0038, Sal-WT vs Sal-KO #*p* = 0.0897. **C** Western Blot analysis for SHANK3 and β-ACTIN of hippocampus P3 fraction. The SHANK3e isoform was analyzed. G:T ****p < 0.0001, G #*p* = 0.0681, Sal-WT vs Sal-Het *p = 0.0429, Sal-WT vs Sal-KO **p = 0.0020, Sal-WT vs Pol-WT *p = 0.0484, Sal-WT vs Pol-Het #*p* = 0.0543, Pol-KO vs Sal-KO **p = 0.0013. **D** Western Blot analysis for SHANK2 and β-ACTIN of hippocampus homogenate. The total SHANK2 was analyzed. G:T **p = 0.0035, G #*p* = 0.0533, T #*p* = 0.0515, Sal-WT vs Sal-Het *p = 0.0317, Sal-WT vs Sal-KO #*p* = 0.0565, Sal-Het vs Pol-KO *p = 0.0172, Sal-KO vs Pol-KO *p = 0.0304. **E** Western Blot analysis for SHANK2 and β-ACTIN of hippocampus P3 fraction. The total SHANK2 was analyzed. G:T ***p = 0.0006, G *p = 0.0309, T *p = 0.0470, Sal-Het vs Pol-KO **p = 0.0042, Sal-KO vs Pol-KO **p = 0.0021, Pol-WT vs Pol-KO *p = 0.0123, Pol-Het vs Pol-KO **p = 0.0082. **F** Western Blot analysis for mGluR5 and β-ACTIN of hippocampus homogenate. **G** Western Blot analysis for mGluR5 and β-ACTIN of hippocampus P3 fraction. G:T ***p = 0.0004, Sal-WT vs Sal-Het *p = 0.0117, Sal-WT vs Sal-KO *p = 0.0124, Sal-WT vs Pol-WT *p = 0.0333, Sal-KO vs Pol-KO *p = 0.0396. **H** Western Blot analysis for HOMER1b/c and β-ACTIN of hippocampus homogenate. T *p = 0.0256, Pol-WT vs Sal-WT *p = 0.0439. **I** Western Blot analysis for HOMER1b/c and β-ACTIN of hippocampus P3 fraction. G:T ****p < 0.0001, G **p = 0.0010, Sal-WT vs Sal-Het ***p = 0.0007, Sal-WT vs Sal-KO ***p = 0.0004, Sal-WT vs Pol-WT **p = 0.0012, Sal-WT vs Pol-Het ***p = 0.0003, Sal-KO vs Pol-KO *p = 0.0236. **J** Western Blot analysis for PSD95 and β-ACTIN of hippocampus homogenate. T #*p* = 0.0581, Pol-WT vs Sal-WT #*p* = 0.0904. **K** Western Blot analysis for PSD95 and β-ACTIN of hippocampus P3 fraction. **B**–**K** Data were tested for normality with the Shapiro–Wilk test followed by two-way ANOVA with a Bonferroni correction for multiple comparisons. Significance level was set to 0.05 (# < 0.10, *p < 0.05, **p < 0.01, ***p < 0.001, ****p < 0.0001). Mean ± SEM, *n* = 3. *G* Genotype of the offspring; *T* Treatment of the mother; *G:T* interaction between the two factors; and *ns* not significant

In the hippocampus, the same fractionation was performed (Fig. 5A, Additional file 1: Fig S14B and Fig S15D-F), and the SHANK3 expression was strongly affected by both the genotype and the Poly I:C treatment. In homogenate, the total SHANK3 and all isoforms analyzed were reduced in Sal-KO compared to Sal-WT (Additional file 1: Fig S18A and Fig. 5B). SHANK3 total, SHANK3a and SHANK3c/d were also reduced in Pol-KO compared to Pol-WT (Additional file 1: Fig S18A), but SHANK3e was increased in Pol-KO compared to all other groups (Fig. 5B). High changes were also observed in the P3 fraction (Additional file 1: Fig S18B and Fig. 5C). SHANK2 followed the same dynamics as in the striatum with a decrease in Sal-KO and an increase in Pol-KO (Fig. 5D, E), as well as mGluR5 (Fig. 5F, G). HOMER1b/c was decreased in Sal-KO (Fig. 5H, I); especially in the P3 fraction there was a strong decrease observed that got increased back to Sal-WT level upon Poly I:C treatment comparable to SHANK3, SHANK2 and mGluR5. PSD95 was not changed in the hippocampus (Fig. 5J, K).

The PFC, on the other hand, was not that strongly affected (Additional file 1: Fig S20, S21, S22, S14C and S15G-I). Again, we saw an increase in SHANK3e in Pol-KO, but only in the homogenate (Additional file 1: Fig S21B) and not in the P3 fraction (Additional file 1: Fig S21C). SHANK2 was increased in Pol-KO in both homogenate and P3 fraction (Additional file 1: Fig S21D and E), comparable to striatum and hippocampus. However, all the other proteins analyzed were not changed in PFC (Additional file 1: Fig S21F-K).

**Fig. 6:**
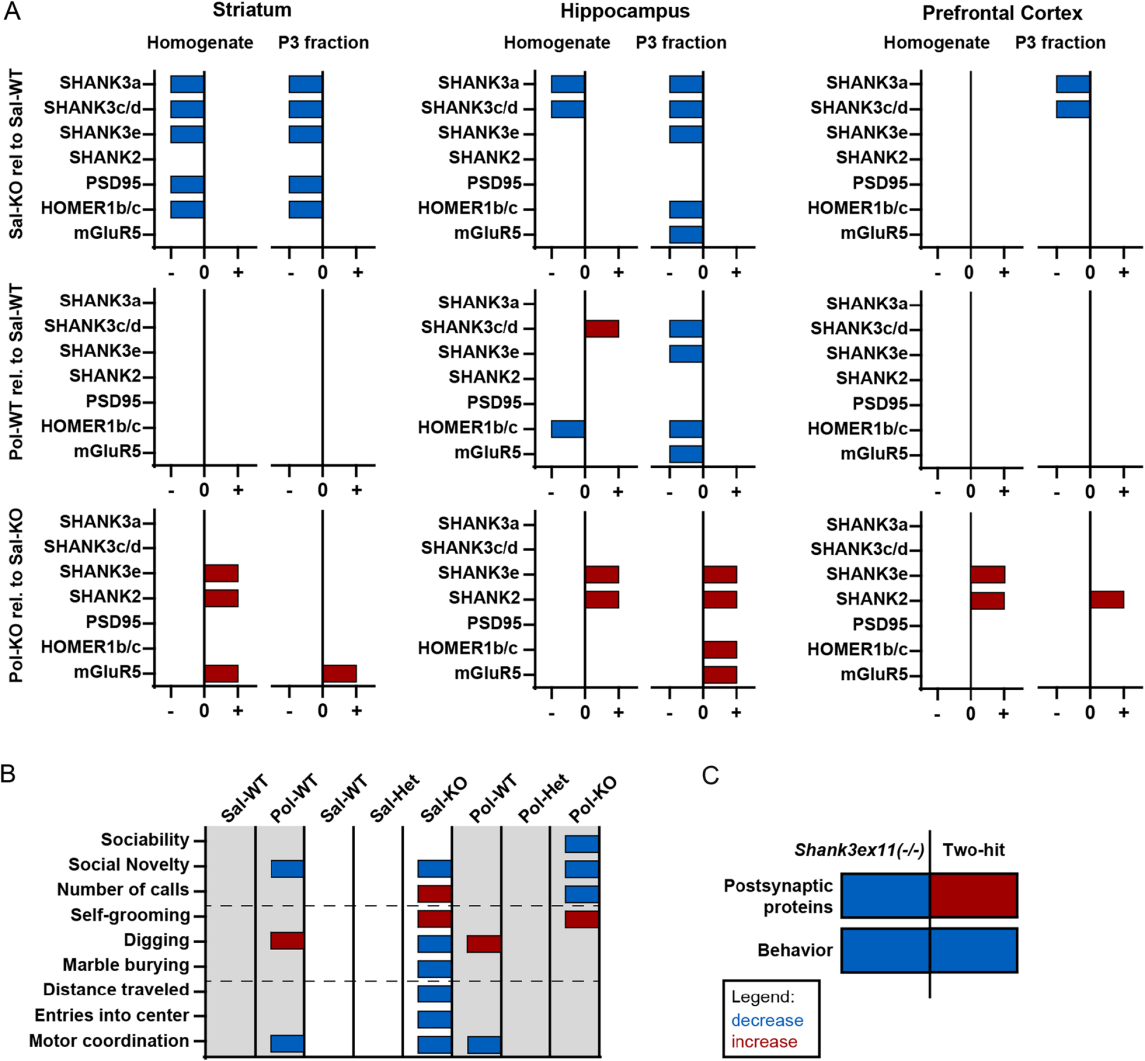
Summary of the results obtained in Western Blot and behavioral analysis. **A** General summary of all Western Blot results, indicating all the proteins affected in the Sal-KO, Pol-WT and Pol-KO groups, first in the striatum, then the hippocampus and finally in the prefrontal cortex. The effects in both the homogenate and the P3 fraction are displayed. The Sal-KO and Pol-WT groups are compared to the control (Sal-WT), whereas Pol-KO is compared to Sal-KO. **B** General summary of all behavioral results, indicating deficits in all experimental groups. All groups are compared to the control (Sal-WT) except Pol-KO which is compared to Sal-KO. The dotted lines separate the three sets of experiments, starting from the top—social behavior, repetitive behavior and comorbidities. **C** Comparison between Sal-KO (*Shank3Δ11 − / −*) and Pol-KO (two-hit), indicating alterations in postsynaptic proteins and behavior. The Sal-KO group is compared to the control (Sal-WT), whereas Pol-KO is compared to Sal-KO. **A**–**C** A red bar indicates an increase, and a blue bar indicates a decrease. The bars are not indicative of the amount of decrease or increase. Only statistically significant results (*p* < 0.05) are displayed in the figure

To summarize the results obtained from Western Blot analysis (Fig. 6A), we compared the Sal-KO to the Sal-WT groups to visualize the findings in the *Shank3Δ11*−*/*− mice. The Sal-KO animals showed a decreased expression of most of the proteins analyzed. The changes were found in homogenate but more substantially in the postsynaptic P3 fraction. Most changes were found in striatum; as reported previously [16], the PFC was affected the least. The Poly I:C injection itself did change the expression of postsynaptic proteins in WT animals (Pol-WT), but solely in the hippocampus. The two-hit mice (comparing Pol-KO to Sal-KO) showed an increase in proteins that previously had been downregulated in the Sal-KO mice. Again, the striatum was affected the most and the PFC the least. Regarding the behavior (Fig. 6B), the *Shank3Δ11*−*/*− mice (Sal-KO) exhibited changes in ASD-like core symptoms and comorbidities, while the Pol-WT group displayed a mild autistic-like phenotype and only an alteration in motor coordination. When comparing the two-hit mice to the Sal-KO, only the core symptoms are aggravated but not the comorbidities. Thus, combining our biochemical and behavioral findings, the two-hit mice show elevated protein expression but compromised behavioral performance (Fig. 6C). Therefore, a genetic or an environmental impact can change the synaptic protein content and, independent of a de- or increase, result in ASD-like behavior.

## Discussion

### The combined influence of *Shank3* deletion and Poly I:C treatment in utero aggravates exclusively specific core symptoms of ASD

Deficits in social interaction and repetitive behavior, the two core symptoms of ASD, but not ASD-associated comorbidities were found to be aggravated in the two-hit mice compared to the single-hit models (*Shank3Δ11*−*/*− or Poly I:C, respectively). Previous studies on *Shank3*-deficient mice have found deficits in sociability [14]; however, other studies indicate no such alteration [50], similar to our results. The two-hit mice were the only group showing a deficit in sociability and social novelty preference. This social approach deficit was confirmed by the same-sex reciprocal social interaction test where the two-hit mice were seldom the ones to initiate the first contact. Thus, the additional Poly I:C in the two-hit mice can unravel an ASD core symptom that was not present in the *Shank3Δ11*−*/*− mice alone. *Shank3*-deficient mice have been challenged with inflammation-inducing stimuli before [51]. Lipopolysaccharide (LPS) injection in juvenile *Shank3* heterozygous mice unmasked social deficits that were not present in saline-injected animals [51]. The authors demonstrated that this was a transient acute reaction to LPS that was not persistent. Our findings showed, for the first time, that a poly I:C-induced challenge as early as in utero can lead to sustainable social deficits in *Shank3*-deficient animals. This hypothesis is supported by the increased time spent self-grooming in the two-hit mice compared to all other groups. The *Shank3Δ11*−*/*− mice also had an increase in the time spent self-grooming as consistently reported in the literature [14, 15, 52, 53], but it was significantly aggravated in the two-hit mice. In our assessment, the WT offspring of poly I:C-injected dams did not display an increase in grooming. This could be due to the fact that bedding material was present in the box and the Pol-WT mice are prone to spend a lot of time digging. Hence, we assessed the time spent digging during the same test and found a significant increase for the Pol-WT mice. Therefore, Pol-WT, Sal-KO and Pol-KO mice all displayed social and repetitive behavioral deficits, but the two-hit mice presented with a more severe phenotype. Regarding the digging behavior of the KO mice, we do not think that we could make the conclusion that this behavior is reduced in the KO mice, as rather than digging, they spent their time grooming. Therefore, this results cannot be interpreted as a decrease interest in digging, but are rather due to the significant increase in grooming.

In social communication, the two-hit mice did not present with deficits in the quantitative parameter number of calls emitted, but emitted fewer calls than the Sal-KO (*Shank3Δ11 − / −)*. The only group that showed a significant increase in USV emission were the *Shank3Δ11*−*/*− mice, a finding also seen exclusively in males by [13]. In this paper, they found, in addition, a reduction in the time spent in contact, whereas we found no differences in time spent in contact and in number of contacts. Nevertheless, the authors have not deprived the tested mice from social contact prior to the test, whereas in our study, we isolated the males four weeks before recording in order to stimulate USV emission as previously recommended [43]. Therefore, the behavior of the *Shank3Δ11*−*/*− mice found in our study could be the result of social isolation. If this is the case, it would mean that the two-hit mice did not react in the same way to the social deprivation. Furthermore, recent studies have focused on recording the animals over longer periods (72 h) and in their natural environment in order to assess spontaneous social communication in its behavioral context without the need to control the motivation of the mice [40, 54, 55]. In our study, *Shank3Δ11*−*/*− and two-hit mice were characterized by emitting more calls ending at a lower frequency (chevron and step down), an untypical finding for the C57BL/6 J mouse strain which are known to emit more upward frequency modulations with jumps [56]. In addition, the WT offspring of poly I:C-injected dams presented with a decrease in the emission of calls with frequency jumps (step up and step down). In summary, the *Shank3Δ11*−*/*− mice (Sal-KO) communicate differently than the Sal-WT mice, and the two-hit group behaves differently from both of them. Regarding social communication, there is an apparent opposing effect between genetic predisposition and environment in the two-hit mice. Hence, the two-hit mice do not present an aggravation of social communication, but rather an alteration. Within the complex scientific field of interaction between genetics and environmental factors that contribute to ASD, it is known that a second hit does not necessarily worsen the symptoms seen in the single-hit models. Thus, it has been described that LPS or fever can ameliorate ASD symptoms [57]. Our two-hit model shows an increase in specific ASD-like core symptoms—self-grooming, sociability and social novelty recognition. Possible underlying mechanisms are discussed in the following paragraph, when discussing the biochemical alterations.

In addition, the two KO groups showed comparable alterations in comorbidities often associated with ASD. The KO groups had a significantly decreased locomotion in the open field test [13, 58]. Nevertheless, they spent the same amount of time in the center zone but made fewer entries which could be due to their hypoactivity. Therefore, KO mice displayed no anxiety-like behavior as was previously found for the *Shank3Δ11*−*/*− model [14]. These groups also displayed strong avoidance behavior during the marble burying test as previously seen [14, 52, 59]. In addition, they also had a deficit in motor coordination during the Rotarod test, which was also present in the Pol-WT mice. The motor phenotype of the KO mice manifests in the open field, the Rotarod and in the grid hanging test, as published previously [60]. It might well be that these motor deficits in part also contribute to the manifestation of other core symptoms of ASD. However, the combined effect of the *Shank3* deletion and the Poly I:C treatment was not present when comorbidities associated with ASD were assessed. The two-hit mice displayed an aggravated phenotype exclusively of the core symptoms of ASD (see also: additonal file 1).

### Biochemical alterations and possible mechanisms

It is well known that *Shank3* deletion in a mouse model decreases the expression of other proteins in the postsynaptic density (PSD). This also reflects in a general decrease in spine density as described for the hippocampus of *Shank3Δex4-9* KO mice by [13], the cortex of *Shank3Δex11* KO mice by [14] and the striatum in Shank3B KO mice [15]. The region mostly affected, as also replicated in our study, is the striatum where a decrease in HOMER1b/c, PSD95 and mGluR5 was found [14, 61, 62]. This goes together with the excessive self-grooming found in those mice, where the striatum is the main region implicated in this behavior [15, 42, 63]. Particularly the levels of HOMER1b/c and mGluR5 were reduced in the hippocampus [13, 64]. Finally, most studies have analyzed the whole cortex, but some have looked specifically in the prefrontal cortex (PFC) and found a decrease in HOMER1b/c expression [65]. Regarding the WT offspring of poly I:C-injected mice, the hippocampus was the most affected region, as found in the literature [66]. However, several studies have analyzed the protein expression of PSD components but mainly in the whole brain [67]. Nevertheless, functional alterations have been found in the hippocampus which were not likely due to presynaptic release properties [25–27]. Moreover, [68] has found a decrease in PSD95 in the hippocampus of adult mice. Indeed, in our study we found a decrease in the expression of HOMER1b/c, mGluR5 and all major isoforms of SHANK3 specifically in the PSD. Therefore, bringing together the behavioral and the biochemical results, the deficit in social novelty recognition in the mice can be explained by these alterations as the ventral hippocampus is involved in the processing of this behavior. As in our study the whole hippocampus was analyzed, we cannot exclude the possibility that cognition is also affected in this two-hit model, as the dorsal hippocampus plays a major role in the processing of cognitive functions such as spatial memory and novel object recognition among others. The two-hit model presented with aggravated core symptoms of autism even though the expression of several PSD proteins was back to normal levels in adulthood. As they still lack the major SHANK3 isoforms, it could be that the structure of the post-synapse is not optimal and does not permit normal functionality of the synapse and therefore this is the reason for the behavioral deficits. Even if in this study we looked into protein expression in distinct brain regions, neural circuit pathology has been found in *Shank3*-deficient mice [69, 70] and in offspring of poly I:C-injected dams [71]. In addition, the interaction between HOMER1 and mGluR5 has been found to be of crucial importance in cortico-striatal connectivity [72, 73].

### The severe reaction of the mother to the Poly I:C injection did not lead to strong offspring deficits

In our study, the pregnant mice reacted severely to the Poly I:C injection as they presented with significant temperature decrease, sickness behavior and a high abortion rate. We would rather have expected an increase in body temperature after injection, since viral infection can cause a fever. Also, previous studies made different observations regarding sickness behavior and the abortion rate. However, a paper that compared several Poly I:C lots from two companies with replication across three laboratories was able to elucidate the reason behind these differences [74]. Some Poly I:C lots contained larger amounts of RNA fragments of high molecular weight which resulted in the dams having a more pronounced immune reaction following the injection. Indeed, in our study we used one of these lots (#117M4005V) and we found the same results—an increased abortion rate and a state of hypothermia. According to our findings, the second lot that we used for the offspring experiments (#118M4035V) had comparable effects and the quality release date of both lots was the same. Therefore, these results point out to the importance of reporting not only the product used but in addition the lot numbers as this could explain potentially non-matching findings and make studies more comparable. For the analysis of the Interleukin 6 concentration in the blood serum of the dams, both WT and Het, another lot was used (#0000112125). Nevertheless, WT and *Shank3* Het dams reacted in a comparable way.

Even though the mothers had a pronounced reaction to the injection of the synthetic virus, the WT offspring presented with a rather mild autistic phenotype and few biochemical alterations. One possible explanation could be the maternal diet. Studies have shown that mice obtained from different suppliers can have very different reactions to Poly I:C and this could be due to the gut microbiome [24]. Indeed, the diet of the mice in our study was particularly rich in zinc as are many commercially available foods. There is evidence of the antiviral activity of zinc [75] and zinc supplementation led to the prevention of autistic-like behaviors in the offspring of Wistar rats injected with LPS [76]. Moreover, the *Shank3* Het mice, offspring of poly I:C-injected dams, did not display an autistic-like phenotype. As these mice have only one copy of the *Shank3* gene, an additional prenatal challenge was expected to lead to deficits. It has been shown that SHANK proteins bind to zinc and their protein levels at the PSD are regulated by zinc concentration [77, 78]. Moreover, in recent years it has been demonstrated that dietary zinc supplementation could prevent ASD-like behaviors in *Shank3*-deficient mice [79]. Therefore, the zinc supplementation could have resulted in a sufficient amount of SHANK2 and SHANK3 being transported to the synapse and this could have prevented the occurrence of an autistic phenotype. This would be in accordance with what we found in the Western Blot analysis, where the *Shank3* heterozygous offspring of saline- or poly I:C-injected dams presented with comparable expression of those proteins.

### Limitations

One limitation of the study is that we can only speculate about possible reasons why the autistic-like phenotype is exacerbated in the two-hit mice. Further analysis might reveal the underlying mechanism. Furthermore, to confirm the hypothesis that the lack of the major isoforms of the SHANK3 protein despite the presence of other postsynaptic proteins is the cause of the behavioral deficits displayed by the two-hit mouse model, isoform-specific SHANK3 antibodies, that are not available so far, would be a great tool. In addition, electrophysiological recordings would have provided information on the functionality of those synapses. Moreover, it might be useful to try other experimental schemes to create a *Shank3* and Poly I:C two-hit. Maybe another injection dose, another time point of injection or repetitive injections could give a more profound ASD-like behavior in WT animals that then can be studied and extended on the *Shank3*-deficient animals. Those dose-dependent experiments might give an even deeper insight into how synaptic protein content after a Poly I:C challenge might ameliorate or aggravate core symptoms of ASD in *Shank3*-deficient mice. Furthermore, behavioral tests focused on assessment of cognition would have made clear if this comorbidity often seen in autism mouse models is also aggravated in the two-hit mice and confirm the conclusion that only core symptoms of autism are exacerbated in the *Shank3Δ11*−*/*− following Poly I:C treatment.

## Conclusions

In summary, male *Shank3Δ11*−*/*− mice, offspring of saline-injected dams, displayed the core symptoms of autism, meaning social approach and communication deficits and increased self-grooming, together with a decrease in the expression of several postsynaptic proteins in all regions analyzed, more pronounced in the PSD (postsynaptic density) than in the whole tissue lysate. Even though the immune reaction of the mother to the Poly I:C injection was severe with an increase in Interleukin 6 in the blood serum and sickness behavior, the wild-type male offspring of those dams showed only a mild autistic-like phenotype and decrease in the expression of postsynaptic proteins exclusively in the hippocampus. Finally, the maternal Poly I:C treatment seemed to have a different effect on the two-hit mice. They displayed a worsening exclusively of the core symptoms of autism, while the levels of several proteins in the PSD were increased compared to the Sal-KO mice. Even though further characterization of the biochemical alterations in the two-hit mouse model is needed to understand the mechanism by which the autistic-like phenotype is exacerbated, this model seems to be a promising tool for the investigation of the interplay between genetic susceptibility and environmental factors in the context of ASD. Environmental factors are also less difficult to manipulate, meaning that treatments can more easily be developed. Future elucidation of the pathways involved in the pathophysiology of autism in this model has the potential to provide possible treatments for ASD-affected individuals.

## Supplementary Information

**Additional file 1.** Supplementary Figures.

## Data Availability

The datasets used and/or analyzed during the current study are available from the corresponding author on reasonable request.

## Abbreviations

ASDs: Autism spectrum disorders
CO_2_: Carbon dioxide
DSM: Diagnostic and Statistical Manual of Mental Disorders
dsRNA: Double-stranded ribonucleic acid
ECL: Enhanced chemiluminescence
EDTA: Ethylenediaminetetraacetic acid
EGTA: Ethylene glycol-bis(β-aminoethyl ether)-*N*,*N*,*N*′,*N*′- tetraacetic acid
ELISA: Enzyme-linked immunosorbent assay
GD: Gestational day
HEPES: 4-(2-Hydroxyethyl)-1-piperazineethanesulfonic acid
Het: Heterozygous, *Shank3*+*/*− mice
HRP: Horseradish peroxidase
Ho: Homogenate
IL-6: Interleukin 6
i.p.: Intraperitoneal
kDa: Kilodalton
kHz: Kilohertz
KO: Knockout, *Shank3−/−* mice
mGluR: Metabotropic glutamate receptor
NaCl: Sodium chloride
NaDOC: Sodium deoxycholate
OF: Open field test
P1: Pellet 1 (nuclei, extracellular matrix and cell debris)
P2: Pellet 2 (crude membrane fraction)
P3: Pellet 3 (Synaptosomes + PSD)
PFC: Prefrontal cortex
PND: Postnatal day
Pol-Het: *Shank3*+*/− *Mice, offspring of a poly I:C-injected dam
Pol-KO: *Shank3−/− *Mice, offspring of a poly I:C-injected dam
Pol-WT: *Shank3*+*/*+ Mice, offspring of a poly I:C-injected dam
Poly I:C: Polyinosinic/polycytidylic acid
PPA: Propionic acid
PSD: Postsynaptic density
PSD95: Postsynaptic density protein 95
Q: Quartile
RNA: Ribonucleic acid
rpm: Revolutions per minute
RSI: Reciprocal social interaction
RT: Room temperature
S1: Supernatant 1 (purified homogenate)
S2: Supernatant 2 (cytosolic compartment)
S3: Supernatant 3 (synaptic cytosol)
Sal-Het: *Shank3*+*/− *Mice, offspring of a saline injected dam
Sal-KO: *Shank3−/− *Mice, offspring of a saline injected dam
Sal-WT: *Shank3*+*/*+ Mice, offspring of a saline injected dam
SDS: Sodium dodecyl sulfate
SDS–PAGE: Sodium dodecyl sulfate–polyacrylamide gel electrophoresis
SEM: Standard error of the mean
SG: Self-grooming
SHANK/ProSAP: SH3 and multiple ankyrin repeat domains protein/Proline-rich synapse-associated proteins
SHANK3: Protein independent of species
SHANK3: Gene (human)
Shank3: Gene (mouse)
SYN: SYNAPTOPHYSIN
TLR-3: Toll-like receptor 3
Tris: Tris-hydroxymethyl aminomethane
USVs: Ultrasonic vocalizations
VPA: Valproic acid
WB: Western Blot
WT: Wild-type, *Shank3*+*/*+ mice
