# Supplementary material for: Immune activation during pregnancy exacerbates ASD-related alterations in Shank3-deficient mice
Source: Mol Autism. 2023 Jan 5;14:1. doi: 10.1186/s13229-022-00532-3 (PMC9814193; doi:10.1186/s13229-022-00532-3)
Supplement: Supplementary file 1 — Additional file 1. Supplementary Figures. [file 13229_2022_532_MOESM1_ESM.docx]

**Supplementary Figures**

**
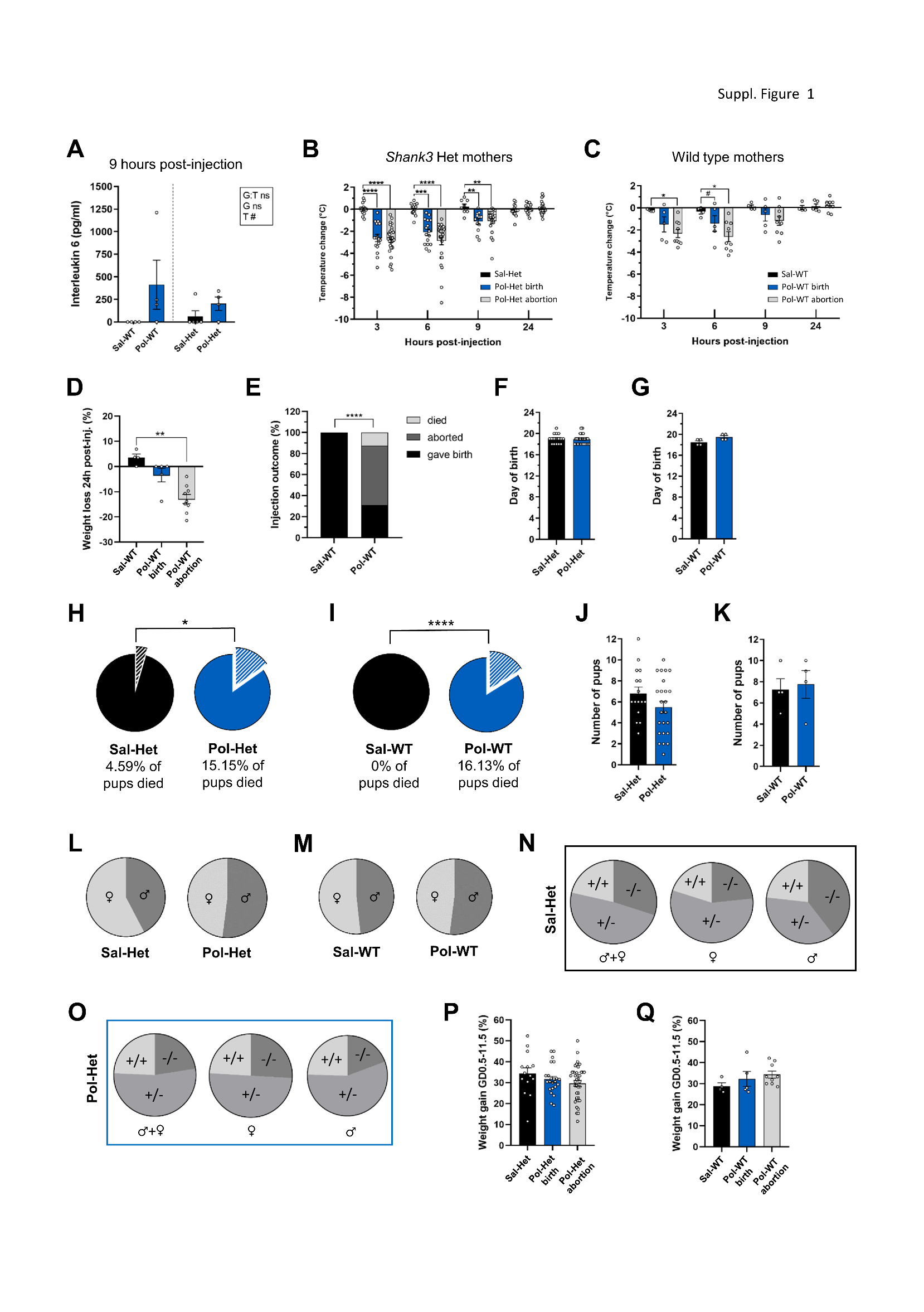
**

**Suppl. Figure 1. Dam response to the injection of the synthetic virus – additional data A** Interleukin 6 blood serum concentration (pg/ml) nine hours post-injection. T #p=0.0645. Data were analyzed by two-way ANOVA followed by a Bonferroni correction for multiple comparisons. Sal-WT n=4, Pol-WT n=4, Sal-Het n=5, Pol-Het n=4. G = Genotype of the mother; T = Treatment of the mother; G:T = interaction between the two factors; ns = not significant. **B** Temperature change (°C) hours post-injection of *Shank3* Het dams. At 3 h – Sal-Het vs Pol-Het birth ****p<0.0001 and Sal-Het vs Pol-Het abortion ****p<0.0001. At 6 h – Sal-Het vs Pol-Het birth ***p=0.0002 and Sal-Het vs Pol-Het abortion ****p<0.0001. At 9 h – Sal-Het vs Pol-Het birth **p=0.0039 and Sal-Het vs Pol-Het abortion **p=0.0047. Sal-Het n=14, Pol-Het birth n=16, Pol-Het abortion n=27. **C** Temperature change (°C) hours post-injection of WT dams. At 3 h – Sal-WT vs Pol-WT abortion *p=0.0208. At 6 h – Sal-WT vs Pol-WT birth #p=0.0851 and Sal-WT vs Pol-WT abortion *p=0.0199. Sal-WT n=4, Pol-WT birth n=5, Pol-WT abortion n=9. **D** Weight lost (%) 24 hours post-injection. Sal-WT vs Pol-WT abortion **p=0.0028. Sal-WT n=4, Pol-WT birth n=5, Pol-WT abortion n=9. **E** Injection outcome (%). Sal-WT vs Pol-WT ****p<0.0005. Data were analyzed by Pearson Chi-square test (expected frequencies ≥ 5), two-sided. Sal-WT n=4, Pol-WT birth n=5, Pol-WT abortion n=9, Pol-WT death n=2. **F** Day of birth of *Shank3* Het dams. Sal-Het n=16 and Pol-Het n=24. **G** Day of birth of WT dams. Sal-WT n=4 and Pol-WT n=4. **H** Pre-weaning mortality of the pups of *Shank3* Het dams. Sal-Het vs Pol-Het *p=0.0320. Sal-Het: 5 died and 104 survived; Pol-Het: 20 died and 112 survived. **I** Pre-weaning mortality of the pups of WT dams. Sal-Het vs Pol-Het ****p<0.0005. Sal-WT: 0 died and 29 survived; Pol-WT: 5 died and 26 survived. **J** Number of pups born to *Shank3* Het dams. Sal-Het n=16 and Pol-Het n=24. **K** Number of pups born to WT dams. Sal-WT n=4 and Pol-WT n=4. **L** Percentage of male and female offspring of *Shank3* Het dams. Sal-Het: 57.69% female and 42.31% male; Pol-Het: 48.65% female and 51.35% male. **M** Percentage of male and female offspring of WT dams. Sal-WT: 51.72% female and 48.28% male; Pol-WT: 48% female and 52% male. **N** Percentage of WT, Het and KO offspring of Sal-Het dams. Females: 20% WT, 23.33% KO and 56.67% Het; Males: 23.26% WT, 39.53% KO and 37.21% Het; Both sexes: 21.36% WT, 30.10% KO and 48.54% Het. **O** Percentage of WT, Het and KO offspring of Pol-Het dams. Females: 24.07% WT, 25.93% KO and 50% Het; Males: 24.56% WT, 19.30% KO and 56.14% Het; Both sexes: 24.32% WT, 22.52% KO and 53.16% Het. **P** Weight gain (%) of *Shank3* Het dams between gestational day (GD) 0.5 and GD11.5. Sal-Het n=15, Pol-Het birth n=23, Pol-Het abortion n=38. **Q** Weight gain (%) of WT dams between gestational day (GD) 0.5 and GD11.5. Sal-WT n=4, Pol-WT birth n=5 and Pol-WT abortion n=9. **B,C,D,P,Q** Data were analyzed by one-way ANOVA followed by a Bonferroni correction for multiple comparisons (when normal) and by Kruskal-Wallis followed by Dunn’s correction for multiple comparisons (when not normal). **F,G,J,K** Data were analyzed by an unpaired two-tailed t-test (when normal) or by Mann-Whitney two-tailed test (when not normal). **H-I** Data were analyzed by Pearson Chi-square test (expected frequencies ≥ 5) or by Fisher’s exact test (expected frequencies < 5), two-sided. **A-Q** Data were tested for normality with the Shapiro-Wilk test. Significance level was set to 0.05 (#<0.10, *p<0.05, **p<0.01, ***p<0.001, ****p<0.0001). Mean ± SEM.

**
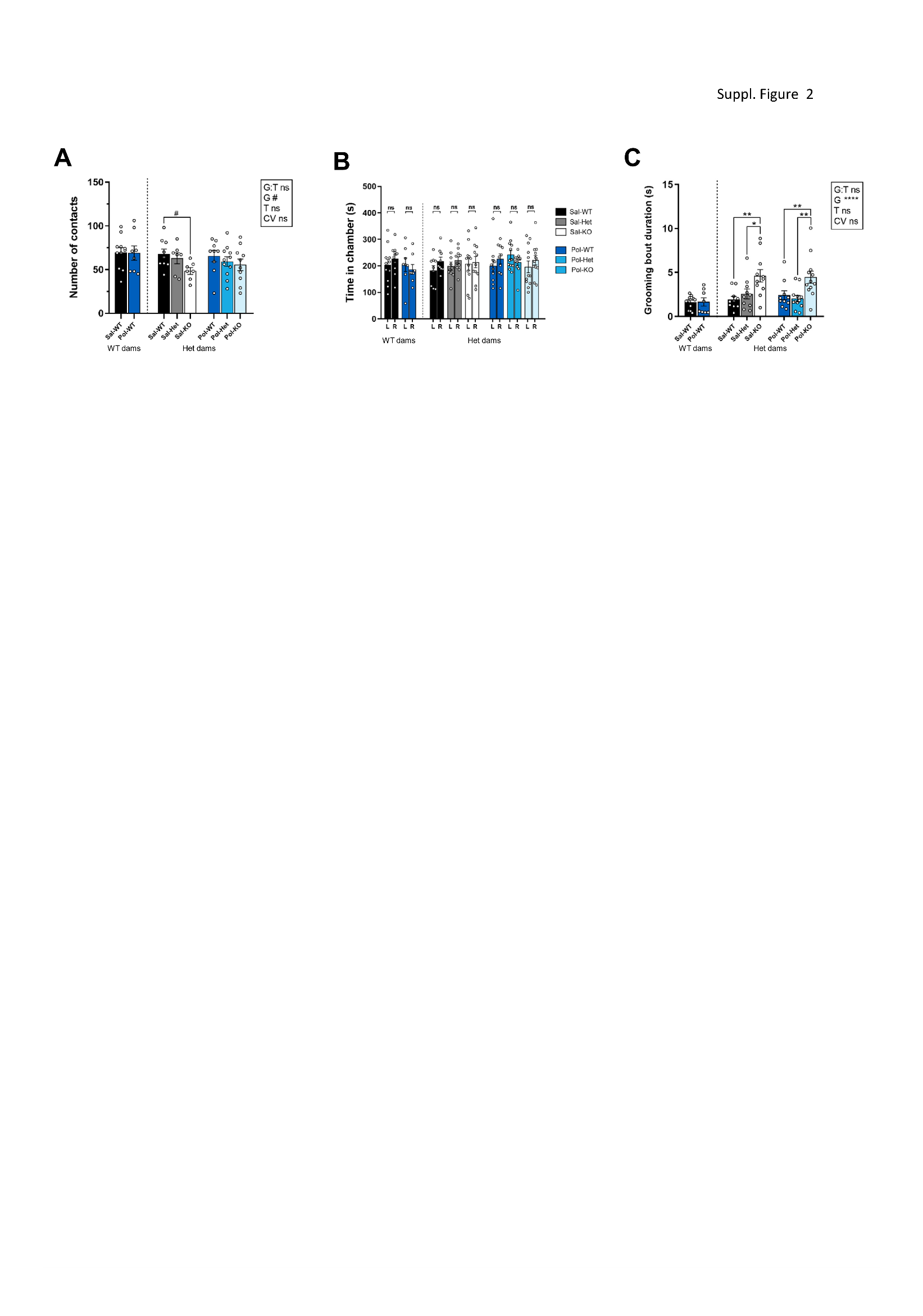
**

**Suppl. Figure 2. Core symptoms of autism – additional data A** Number of contacts made during same-sex reciprocal social interaction. G #p =0.07, Sal-WT vs Sal-KO #p=0.10. Sal-WT (WT dams) n=11, Pol-WT (WT dams) n=8, Sal-WT (Het dams) n=9, Sal-Het n=7, Sal-KO n=7, Pol-WT (Het dams) n=9, Pol-Het n=11, Pol-KO n=10. **B** Time spent in chamber (s) during the habituation phase of the three chambers test. Data were analyzed by a paired two-tailed t-test (when normal) or by Wilcoxon matched-pairs signed rank two-tailed test (when not normal). Sal-WT (WT dams) n=11, Pol-WT (WT dams) n=8, Sal-WT (Het dams) n=9, Sal-Het n=10, Sal-KO n=11, Pol-WT (Het dams) n=10, Pol-Het n=12, Pol-KO n=12. L = Left chamber, R = Right chamber. **C** Grooming bout duration (s). G ****p<0.0005, Sal-KO vs the following: Sal-WT **p=0.0010 and Sal-Het *p=0.0170. Pol-KO vs the following: Pol-WT **p=0.0050 and Pol-Het **p=0.0020. Sal-WT (WT dams) n=12, Pol-WT (WT dams) n=8, Sal-WT (Het dams) n=10, Sal-Het n=10, Sal-KO n=11, Pol-WT (Het dams) n=10, Pol-Het n=12, Pol-KO n=12. **A,C** Data were analyzed by two-way ANCOVA followed by a Bonferroni correction for multiple comparisons. G = Genotype of the offspring; T = Treatment of the mother; G:T = interaction between the two factors; CV = covariate “Genotype of the mother”. **A-C** Data were tested for normality with the Shapiro-Wilk test. Significance level was set to 0.05 (#<0.10, *p<0.05, **p<0.01, ***p<0.001, ****p<0.0001). Mean ± SEM. ns = not significant.

**
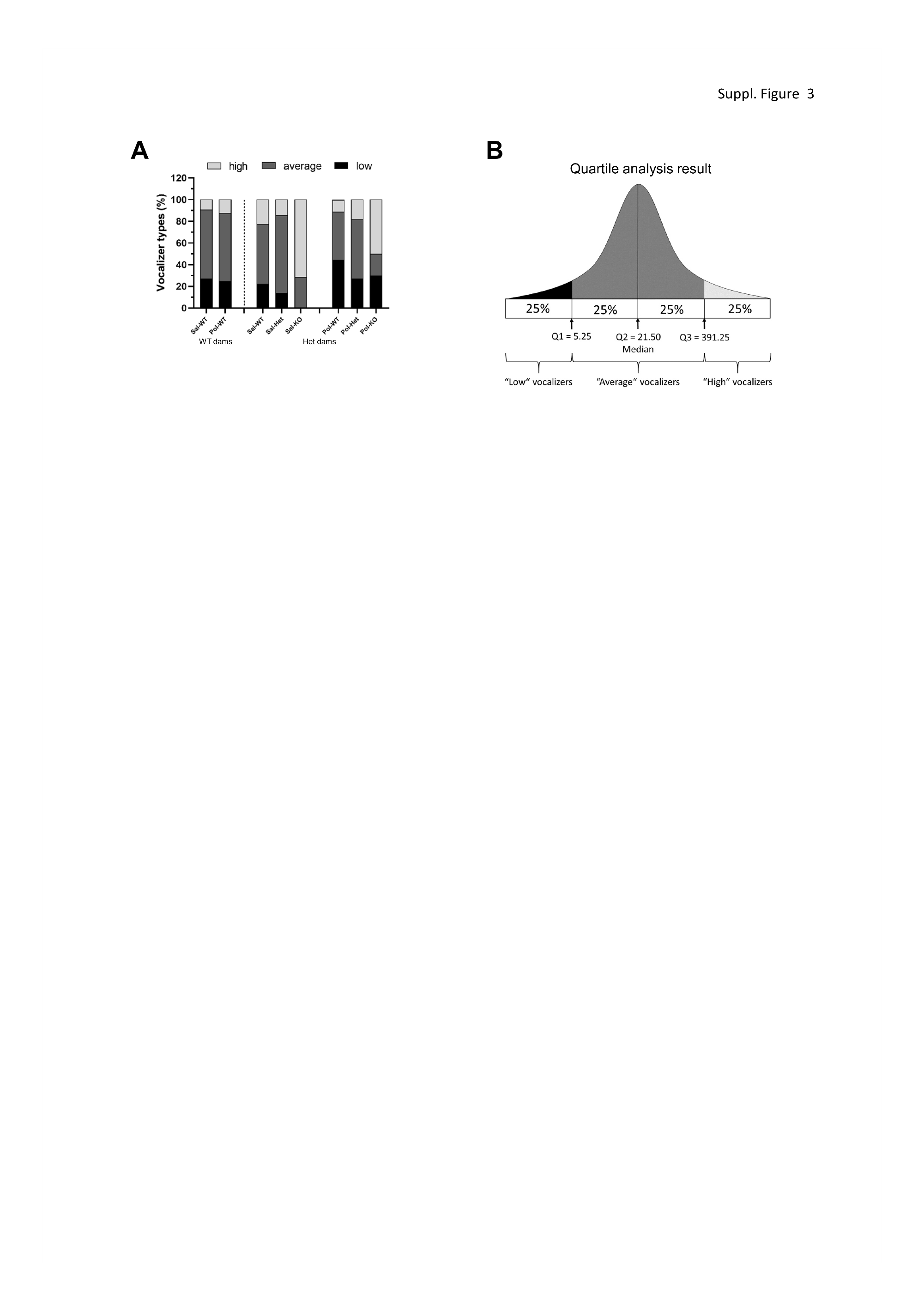
**

**Suppl. Figure 3. Deficits in ultrasonic vocalizations – additional data A** Percentage of vocalizer types during same-sex reciprocal social interaction. Data were analyzed by pairwise Fisher’s exact test followed by a Benjamini-Hochberg correction for multiple comparisons. **B** Quartile analysis of the total number of calls emitted during same-sex reciprocal social interaction. **A-B** Data were tested for normality with the Shapiro-Wilk test. Significance level was set to 0.05 (#<0.10, *p<0.05, **p<0.01, ***p<0.001, ****p<0.0001). Mean ± SEM. Sal-WT (WT dams) n=11, Pol-WT (WT dams) n=8, Sal-WT (Het dams) n=9, Sal-Het n=7, Sal-KO n=7, Pol-WT (Het dams) n=9, Pol-Het n=11, Pol-KO n=10.

**
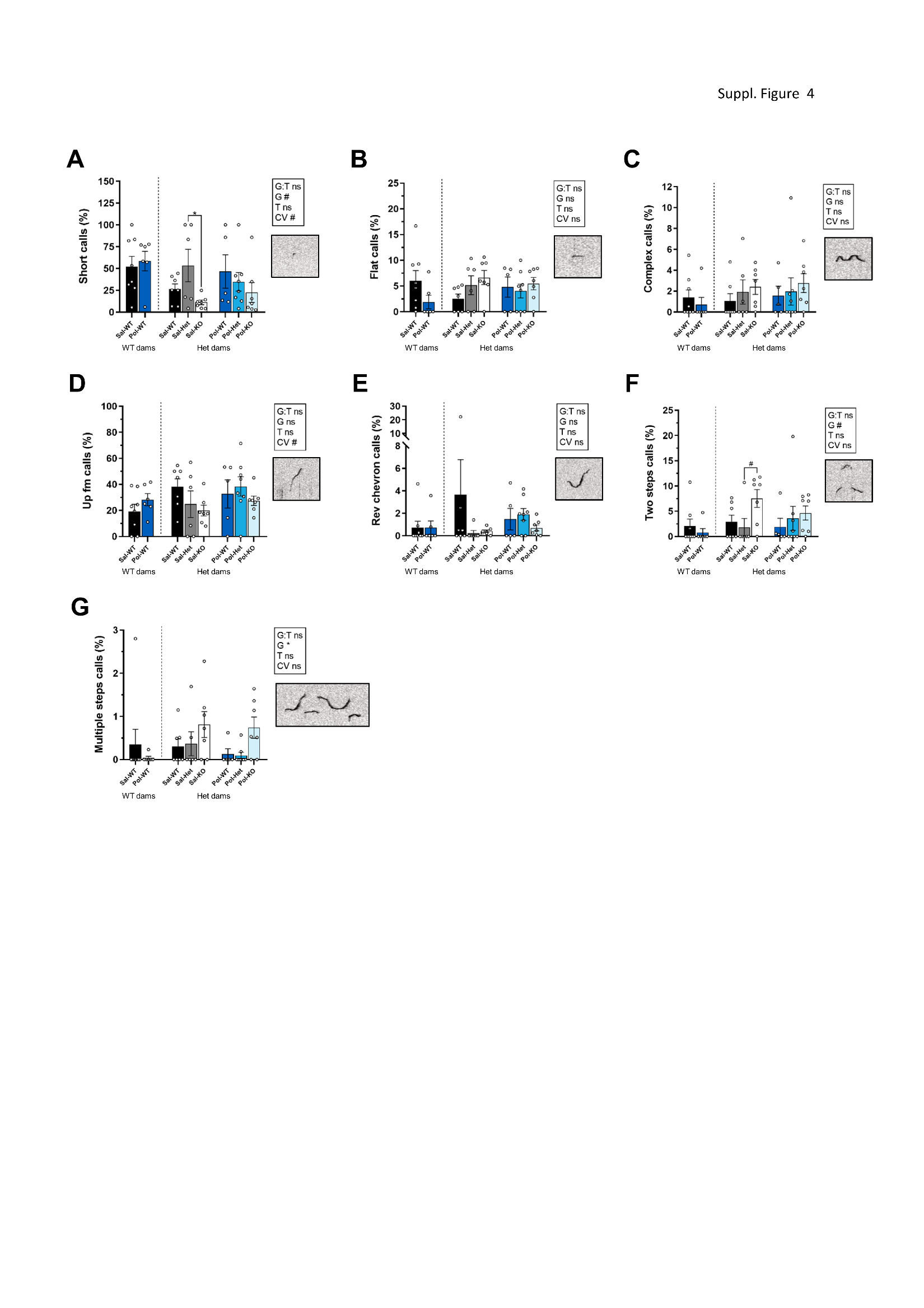
**

**Suppl. Figure 4. Ultrasonic vocalization types – additional data A** Percentage of short calls. G #p=0.0620, CV #p=0.0990, Sal-KO vs Sal-Het *p=0.0460. **B** Percentage of flat calls. **C** Percentage of complex calls. **D** Percentage of up fm calls. CV #p=0.0660. fm = frequency modulation. **E** Percentage of rev chevron calls. rev = reverse. **F** Percentage of two steps calls. G #p=0.0580, Sal-KO vs Sal-Het #p=0.0650. **G** Percentage of multiple steps calls. G *p=0.0300. **A-G** Data were tested for normality with the Shapiro-Wilk test followed by two-way ANCOVA with a Bonferroni correction for multiple comparisons. Significance level was set to 0.05 (#<0.10, *p<0.05, **p<0.01, ***p<0.001, ****p<0.0001). Mean ± SEM. Sal-WT (WT dams) n=11, Pol-WT (WT dams) n=8, Sal-WT (Het dams) n=9, Sal-Het n=7, Sal-KO n=7, Pol-WT (Het dams) n=9, Pol-Het n=11, Pol-KO n=10. G = Genotype of the offspring; T = Treatment of the mother; G:T = interaction between the two factors; CV = covariate “Genotype of the mother”, ns = not significant.


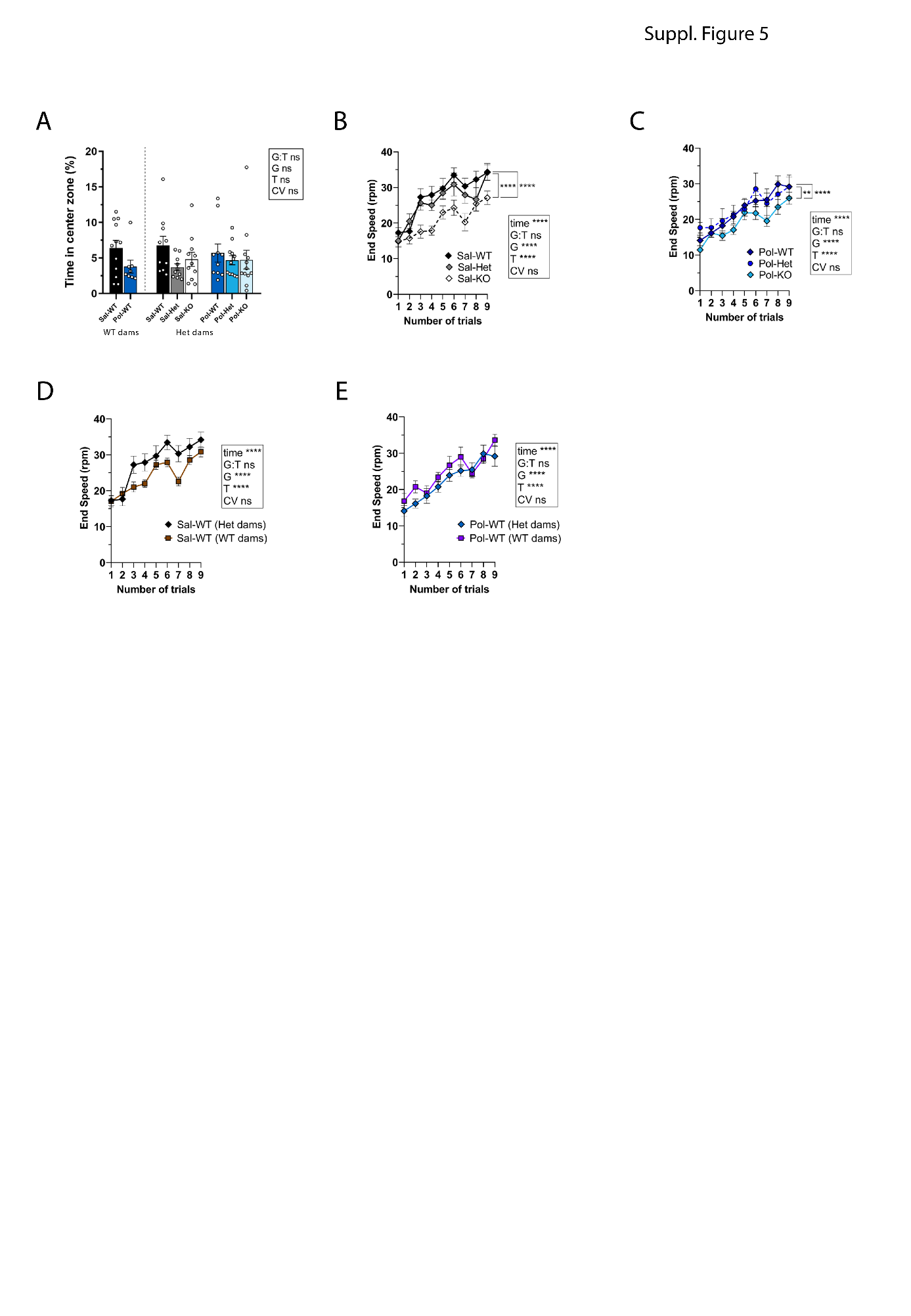


**Suppl. Figure 5. Comorbidities – additional data A** Time spent in the center zone of the open field arena (s). Data were analyzed by two-way ANCOVA followed by a Bonferroni correction for multiple comparisons. Sal-WT (WT dams) n=12, Pol-WT (WT dams) n=8, Sal-WT (Het dams) n=10, Sal-Het n=10, Sal-KO n=11, Pol-WT (Het dams) n=10, Pol-Het n=12, Pol-KO n=12. **B-E** End speed (rpm) during the Rotarod test. G:T ns, time ****p<0.0001, G ****p<0.0001 and T ****p<0.0001, Sal-KO vs the following: Sal-Het ****p<0.0001 and Sal-WT ****p<0.0001. Pol-KO vs the following: Pol-Het **p=0.0024 and Pol-WT ****p<0.0001. Sal-WT (Het dams) vs Sal-WT (WT dams) and Pol-WT (Het dams) vs Pol-WT (WT dams) – no significant differences, CV (covariate, genotype of the mother) p=0.1134. Data were analyzed by a linear mixed model analysis with repeated measures followed by a Tukey-Kramer correction for multiple comparisons. Sal-WT (WT dams) n=12, Pol-WT (WT dams) n=8, Sal-WT (Het dams) n=10, Sal-Het n=8, Sal-KO n=11, Pol-WT (Het dams) n=10, Pol-Het n=6, Pol-KO n=12. **A-C** Data were tested for normality with the Shapiro-Wilk test. Significance level was set to 0.05 (#<0.10, *p<0.05, **p<0.01, ***p<0.001, ****p<0.0001). Mean ± SEM. G = Genotype of the offspring; T = Treatment of the mother; G:T = interaction between the two factors; CV = covariate “Genotype of the mother”, ns = not significant.


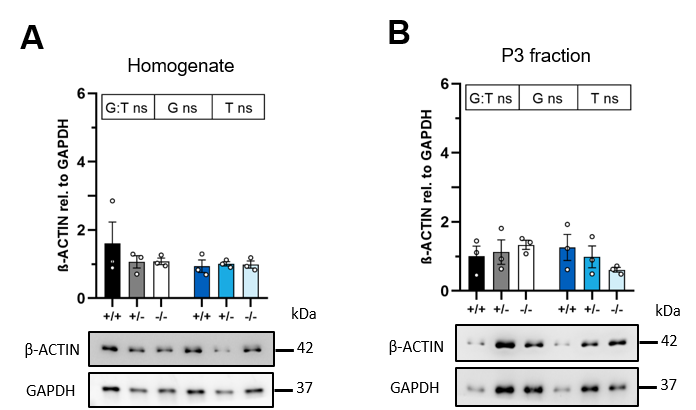


**Suppl. Figure 6. Western Blot – loading control test A** In the homogenate of the prefrontal cortex, the expression of ß-ACTIN (the loading control used in this study) was normalized over another possible loading control – GAPDH. **B** In the P3 fraction of the prefrontal cortex, the expression of ß-ACTIN (the loading control used in this study) was normalized over another possible loading control – GAPDH. **A-B** Data were tested for normality with the Shapiro-Wilk test followed by two-way ANOVA with a Bonferroni correction for multiple comparisons. Significance level was set to 0.05 (#<0.10, *p<0.05, **p<0.01, ***p<0.001, ****p<0.0001). Mean ± SEM, n=3. G = Genotype of the offspring; T = Treatment of the mother; G:T = interaction between the two factors; ns = not significant.

**
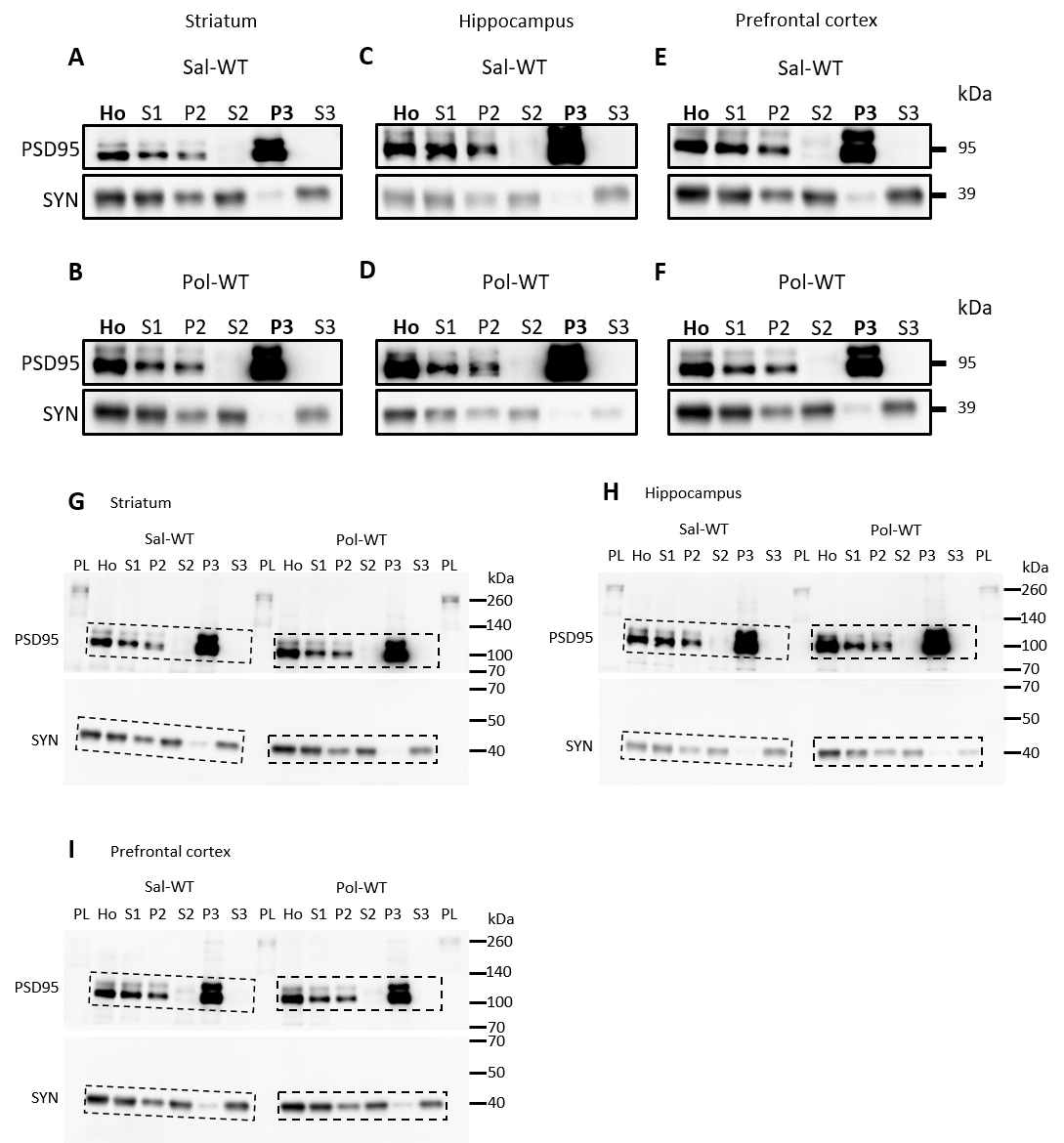
**

**Suppl. Figure 7. Subcellular fractionation of the brain tissue of the offspring of WT dams. A** Fractionation in the striatum of Sal-WT offspring. Western Blot for PSD95 (postsynaptic) and SYNAPTOPHYSIN (SYN, presynaptic)**. B** Fractionation in the striatum of Pol-WT offspring. Western Blot for PSD95 (postsynaptic) and SYNAPTOPHYSIN (SYN, presynaptic). **C** Fractionation in the hippocampus of Sal-WT offspring. Western Blot for PSD95 (postsynaptic) and SYNAPTOPHYSIN (SYN, presynaptic). **D** Fractionation in the hippocampus of Pol-WT offspring. Western Blot for PSD95 (postsynaptic) and SYNAPTOPHYSIN (SYN, presynaptic). **E** Fractionation in the PFC of Sal-WT offspring. Western Blot for PSD95 (postsynaptic) and SYNAPTOPHYSIN (SYN, presynaptic). **F** Fractionation in the PFC of Pol-WT offspring. Western Blot for PSD95 (postsynaptic) and SYNAPTOPHYSIN (SYN, presynaptic). **G** Fractionation in the striatum of Sal-WT and Pol-WT offspring. Western Blot for PSD95 (postsynaptic) and SYNAPTOPHYSIN (SYN, presynaptic) – uncropped blots**. H** Fractionation in the hippocampus of Sal-WT and Pol-WT offspring. Western Blot for PSD95 (postsynaptic) and SYNAPTOPHYSIN (SYN, presynaptic) – uncropped blots**. I** Fractionation in the PFC of Sal-WT and Pol-WT offspring. Western Blot for PSD95 (postsynaptic) and SYNAPTOPHYSIN (SYN, presynaptic) - uncropped blots. **A-I** Protein ladder used – Spectra Broad Range.


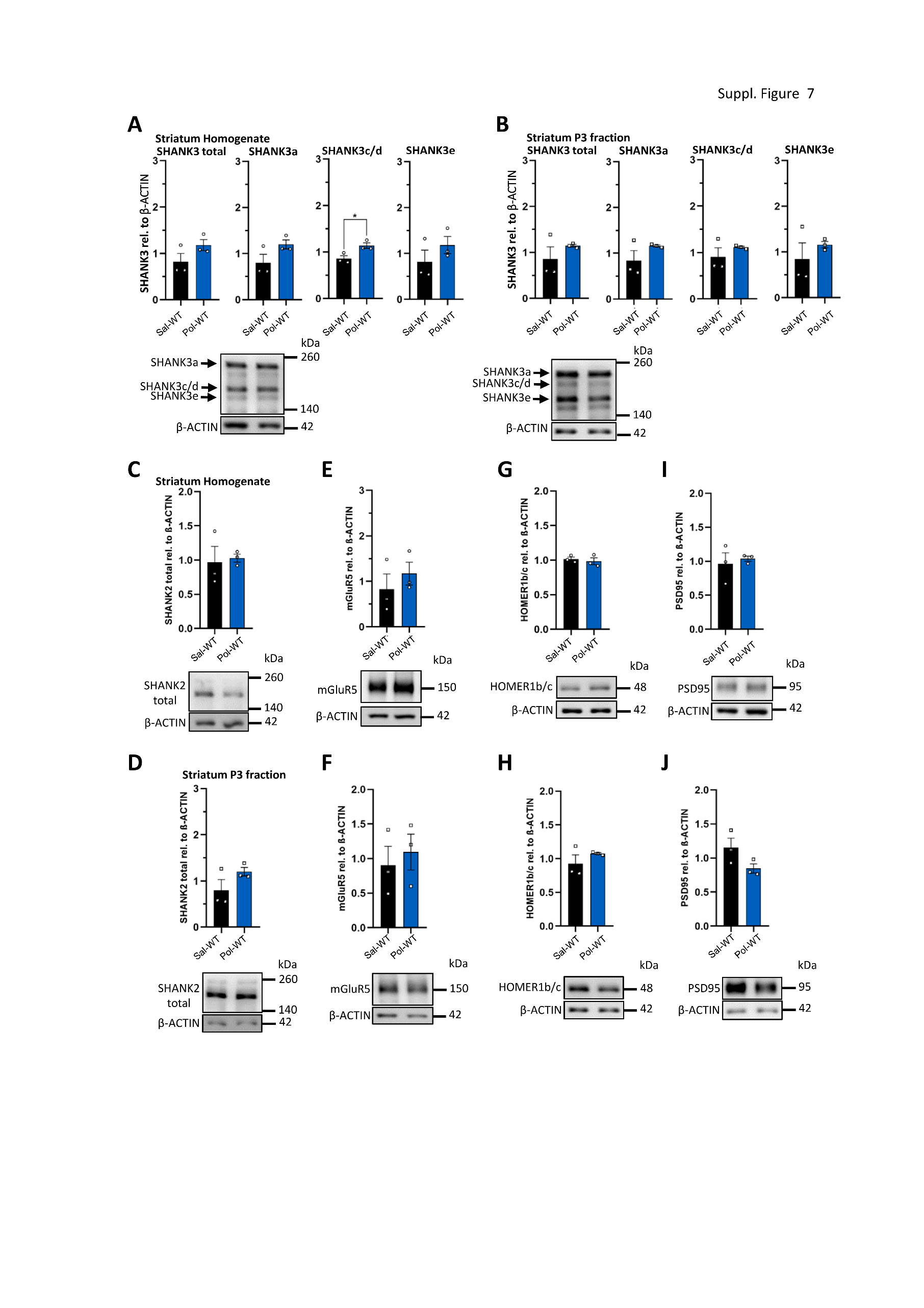


**Suppl. Figure 8. Synaptic changes in striatum of the offspring of WT dams. A+B** Western Blot analysis for SHANK3 and β-ACTIN in homogenate (**A**) and in the P3 fraction (**B**). Pol-WT vs Sal-WT *p=0.0300. **C+D** Western Blot analysis for SHANK2 and β-ACTIN in homogenate (**C**) and in the P3 fraction (**D**). **E+F** Western Blot analysis for mGluR5 and β-ACTIN in homogenate (**E**) and in the P3 fraction (**F**). **G+H** Western Blot analysis for HOMER1b/c and β-ACTIN in homogenate (**G**) and in the P3 fraction (**H**). **I+J** Western Blot analysis for PSD95 and β-ACTIN in homogenate (**I**) and in the P3 fraction (**J**). **A-J** Data were tested for normality with the Shapiro-Wilk test followed by an unpaired two-tailed t-test (when normal) or by Mann-Whitney two-tailed test (when not normal). Significance level was set to 0.05 (#<0.10, *p<0.05, **p<0.01, ***p<0.001, ****p<0.0001). Mean ± SEM, n=3. ns = not significant.


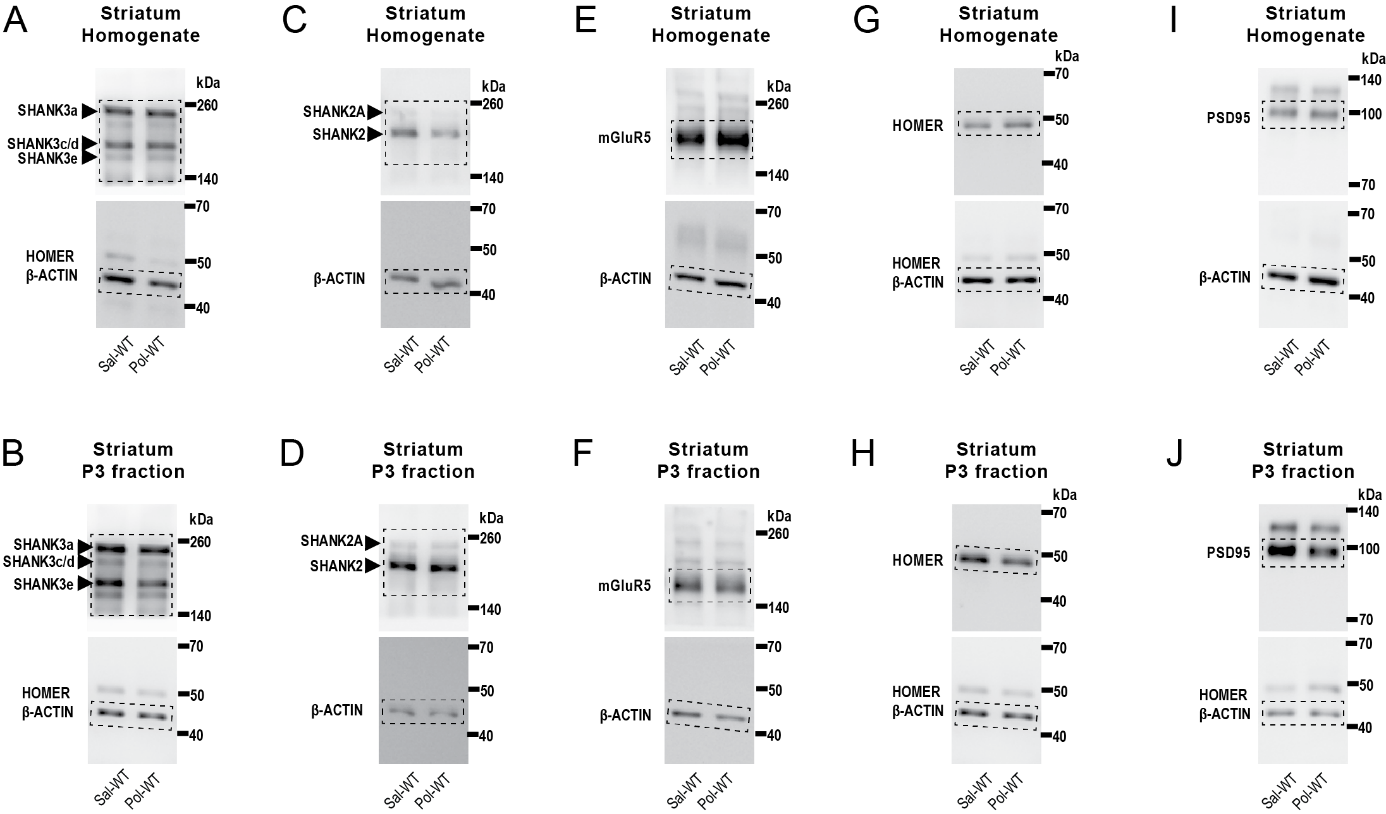


**Suppl. Figure 9. All uncropped Western Blots - striatum of the offspring of WT dams. A+B** Uncropped Western Blots of SHANK3 and β-ACTIN in homogenate (**A**) and in the P3 fraction (**B**). **C+D** Uncropped Western Blots of SHANK2 and β-ACTIN in homogenate (**C**) and in the P3 fraction (**D**). **E+F** Uncropped Western Blots of mGluR5 and β-ACTIN in homogenate (**E**) and in the P3 fraction (**F**). **G+H** Uncropped Western Blots of HOMER and β-ACTIN in homogenate (**G**) and in the P3 fraction (**H**). **I+J** Uncropped Western Blots of PSD95 and β-ACTIN in homogenate (**I**) and in the P3 fraction (**J**). **A-J** On several blots HOMER can be seen next to ß-ACTIN, this is due to the fact that sometimes HOMER was detected first and then the membrane was incubated with ß-ACTIN again over night.

**
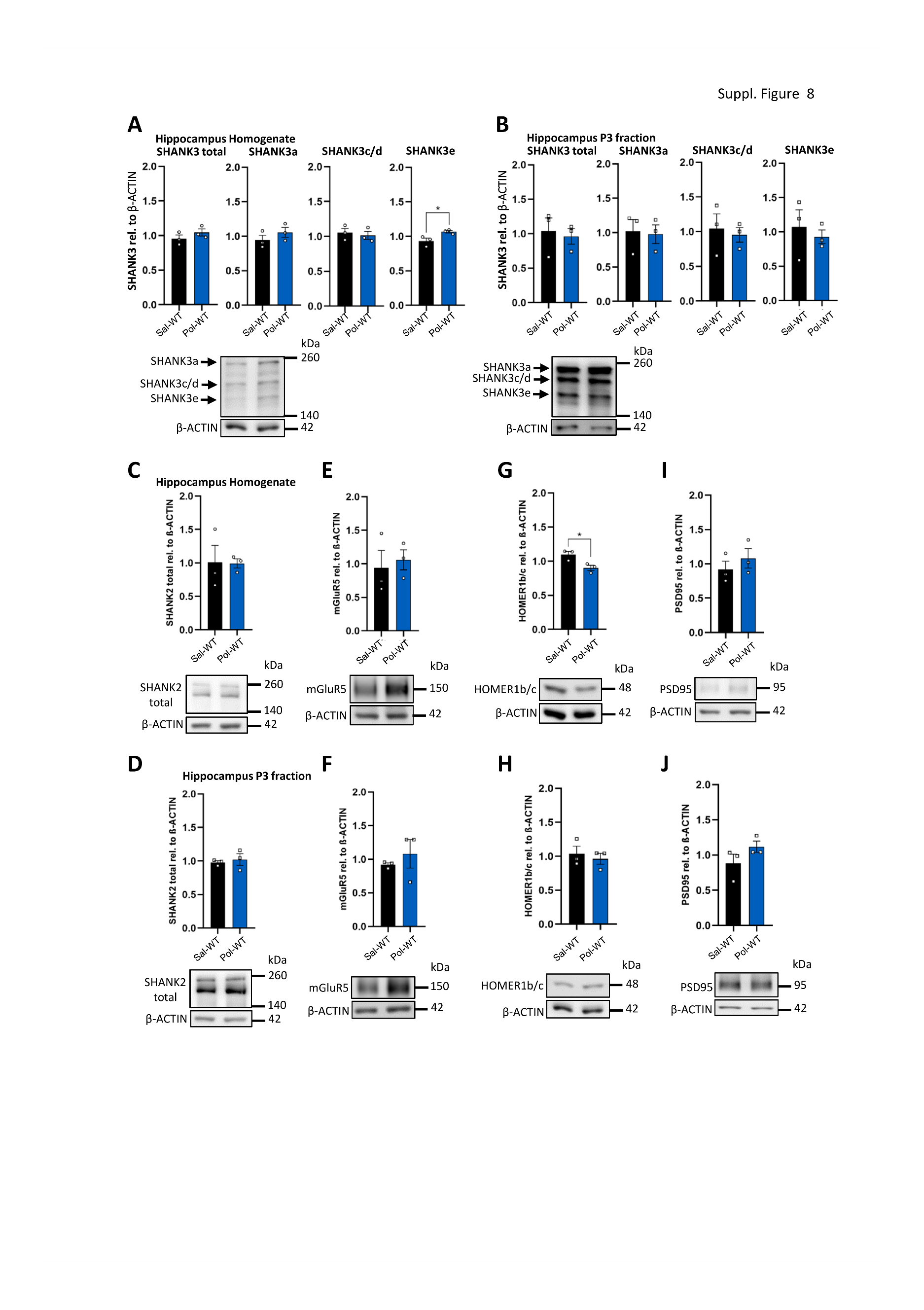
**

**Suppl. Figure 10. Synaptic changes in hippocampus of the offspring of WT dams. A+B** Western Blot analysis for SHANK3 and β-ACTIN in homogenate (**A**) and in the P3 fraction (**B**). Pol-WT vs Sal-WT *p=0.0414. **C+D** Western Blot analysis for SHANK2 and β-ACTIN in homogenate (**C**) and in the P3 fraction (**D**). **E+F** Western Blot analysis for mGluR5 and β-ACTIN in homogenate (**E**) and in the P3 fraction (**F**). **G+H** Western Blot analysis for HOMER1b/c and β-ACTIN in homogenate (**G**) and in the P3 fraction (**H**). Sal-WT vs Pol-WT *p=0.0302. **I+J** Western Blot analysis for PSD95 and β-ACTIN in homogenate (**I**) and in the P3 fraction (**J**). **A-J** Data were tested for normality with the Shapiro-Wilk test followed by an unpaired two-tailed t-test (when normal) or by Mann-Whitney two-tailed test (when not normal). Significance level was set to 0.05 (#<0.10, *p<0.05, **p<0.01, ***p<0.001, ****p<0.0001). Mean ± SEM, n=3. ns = not significant.


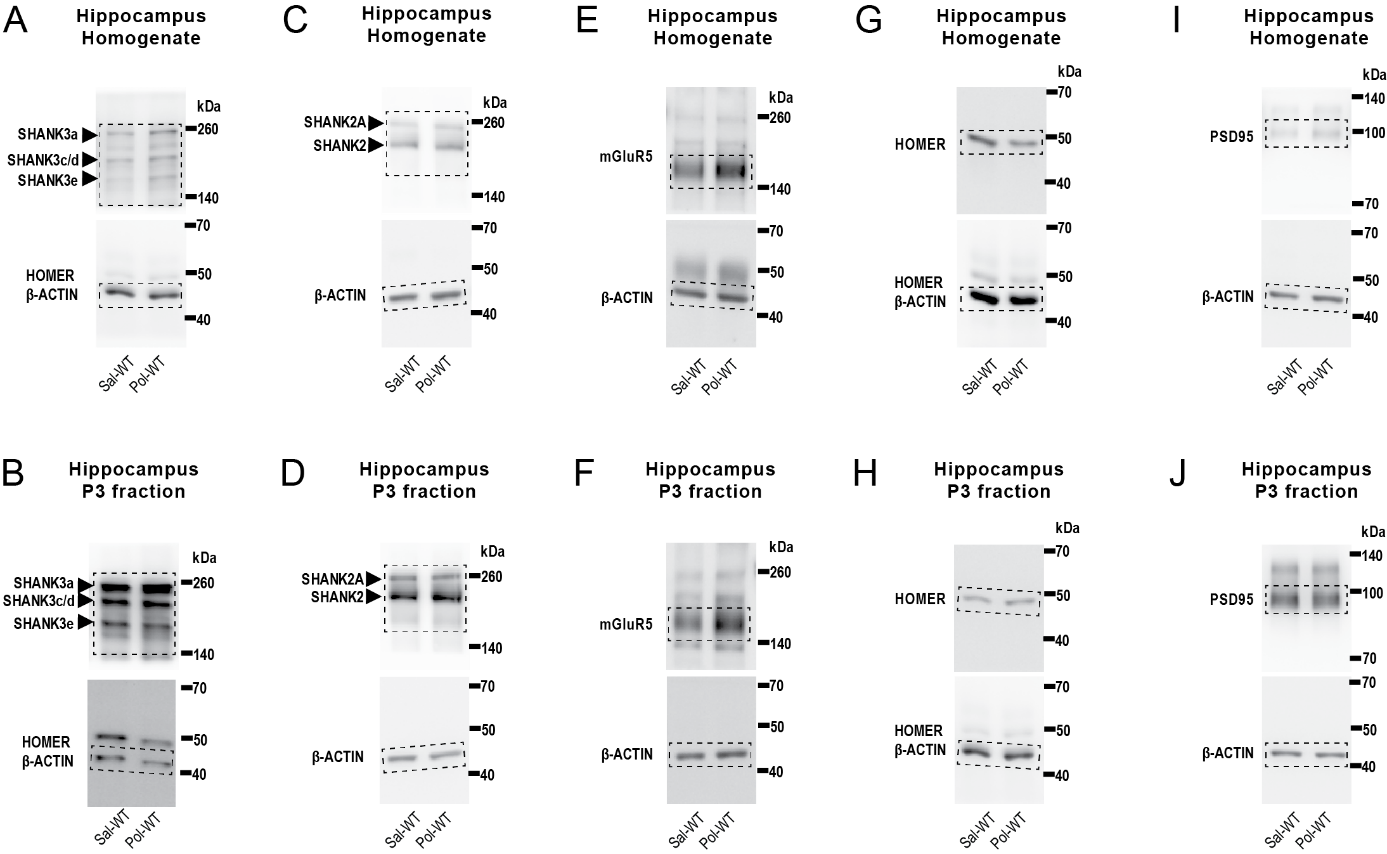


**Suppl. Figure 11. All uncropped Western Blots - hippocampus of the offspring of WT dams. A+B** Uncropped Western Blots of SHANK3 and β-ACTIN in homogenate (**A**) and in the P3 fraction (**B**). **C+D** Uncropped Western Blots of SHANK2 and β-ACTIN in homogenate (**C**) and in the P3 fraction (**D**). **E+F** Uncropped Western Blots of mGluR5 and β-ACTIN in homogenate (**E**) and in the P3 fraction (**F**). **G+H** Uncropped Western Blots of HOMER and β-ACTIN in homogenate (**G**) and in the P3 fraction (**H**). **I+J** Uncropped Western Blots of PSD95 and β-ACTIN in homogenate (**I**) and in the P3 fraction (**J**). **A-J** On several blots HOMER can be seen next to ß-ACTIN, this is due to the fact that sometimes HOMER was detected first and then the membrane was incubated with ß-ACTIN again over night.

**
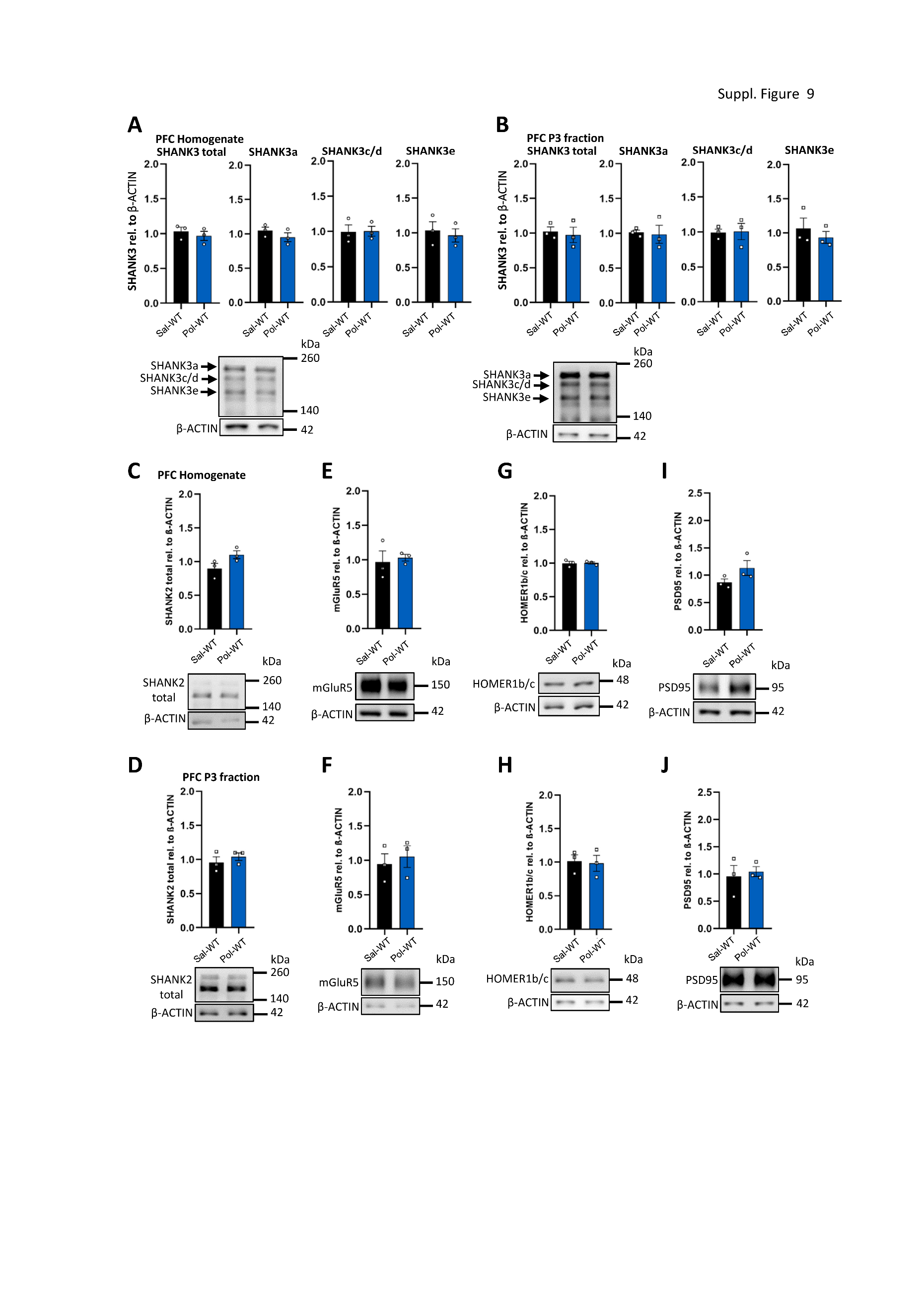
**

**Suppl. Figure 12. Synaptic changes in PFC of the offspring of WT dams. A+B** Western Blot analysis for SHANK3 and β-ACTIN in homogenate (**A**) and in the P3 fraction (**B**). **C+D** Western Blot analysis for SHANK2 and β-ACTIN in homogenate (**C**) and in the P3 fraction (**D**). **E+F** Western Blot analysis for mGluR5 and β-ACTIN in homogenate (**E**) and in the P3 fraction (**F**). **G+H** Western Blot analysis for HOMER1b/c and β-ACTIN in homogenate (**G**) and in the P3 fraction (**H**). **I+J** Western Blot analysis for PSD95 and β-ACTIN in homogenate (**I**) and in the P3 fraction (**J**). **A-J** Data were tested for normality with the Shapiro-Wilk test followed by an unpaired two-tailed t-test (when normal) or by Mann-Whitney two-tailed test (when not normal). Significance level was set to 0.05 (#<0.10, *p<0.05, **p<0.01, ***p<0.001, ****p<0.0001). Mean ± SEM, n=3. ns = not significant.


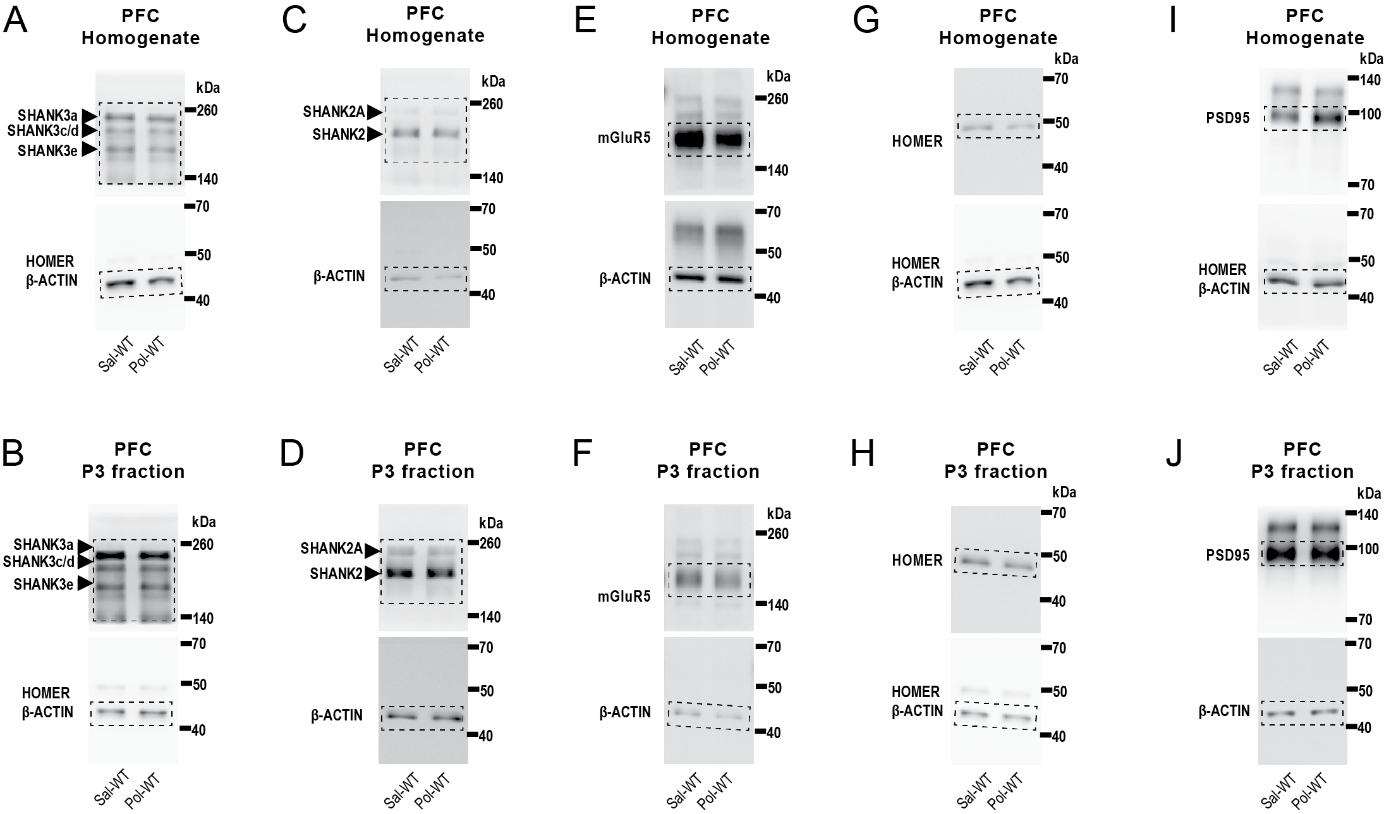


**Suppl. Figure 13. All uncropped Western Blots – prefrontal cortex (PFC) of the offspring of WT dams. A+B** Uncropped Western Blots of SHANK3 and β-ACTIN in homogenate (**A**) and in the P3 fraction (**B**). **C+D** Uncropped Western Blots of SHANK2 and β-ACTIN in homogenate (**C**) and in the P3 fraction (**D**). **E+F** Uncropped Western Blots of mGluR5 and β-ACTIN in homogenate (**E**) and in the P3 fraction (**F**). **G+H** Uncropped Western Blots of HOMER and β-ACTIN in homogenate (**G**) and in the P3 fraction (**H**). **I+J** Uncropped Western Blots of PSD95 and β-ACTIN in homogenate (**I**) and in the P3 fraction (**J**). **A-J** On several blots HOMER can be seen next to ß-ACTIN, this is due to the fact that sometimes HOMER was detected first and then the membrane was incubated with ß-ACTIN again over night.

**
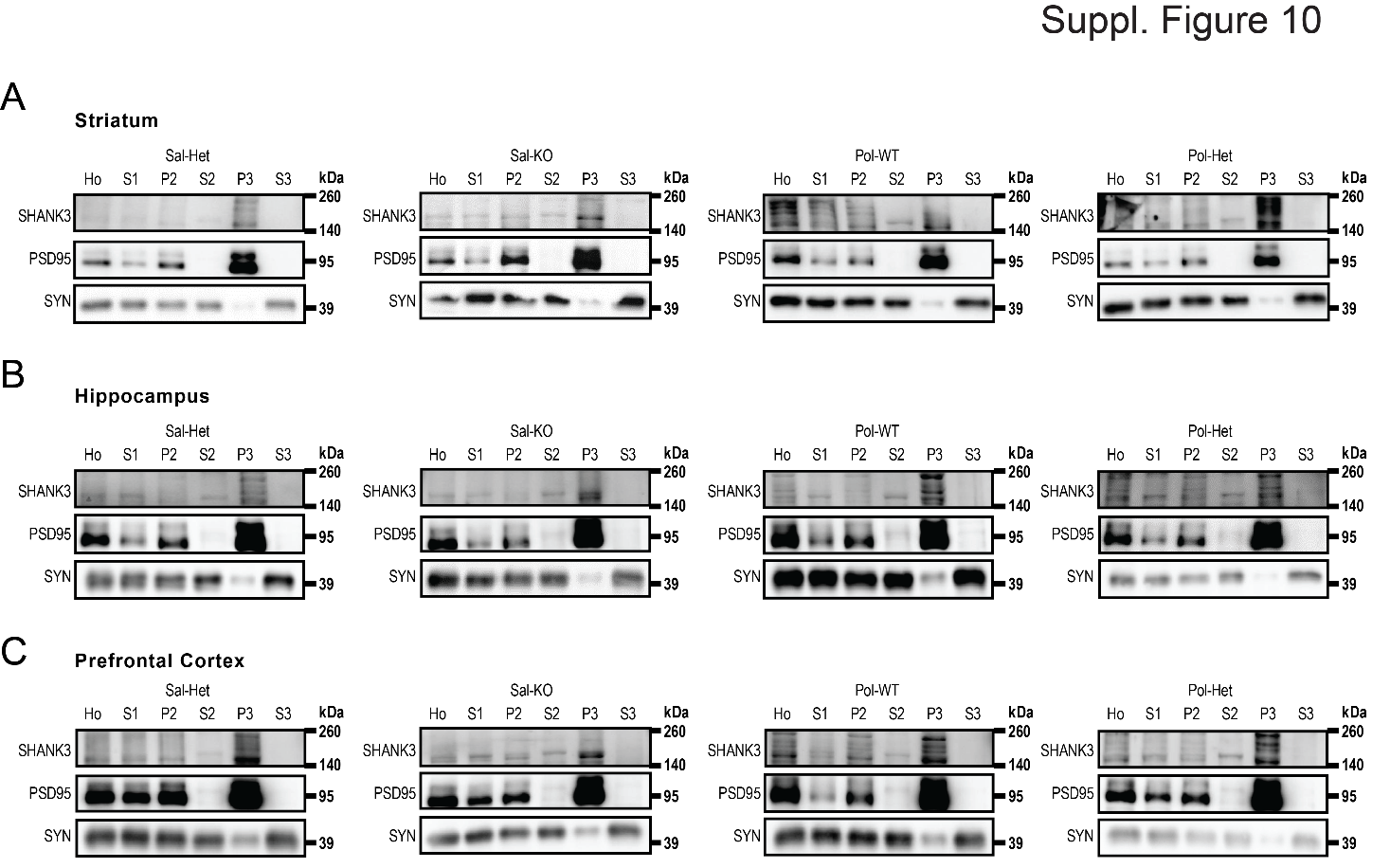
**

**Suppl. Figure 14. Subcellular fractionation of the brain tissue of the offspring of Het dams. A** Fractionation in the striatum of Sal-Het, Sal-KO, Pol-WT and Pol-Het. Western Blot for SHANK3, PSD95 (postsynaptic) and SYNAPTOPHYSIN (SYN, presynaptic)**. B** Fractionation in the hippocampus of Sal-Het, Sal-KO, Pol-WT and Pol-Het. Western Blot for SHANK3, PSD95 (postsynaptic) and SYNAPTOPHYSIN (SYN, presynaptic)**. C** Fractionation in the PFC of Sal-Het, Sal-KO, Pol-WT and Pol-Het. Western Blot for SHANK3, PSD95 (postsynaptic) and SYNAPTOPHYSIN (SYN, presynaptic)**.**


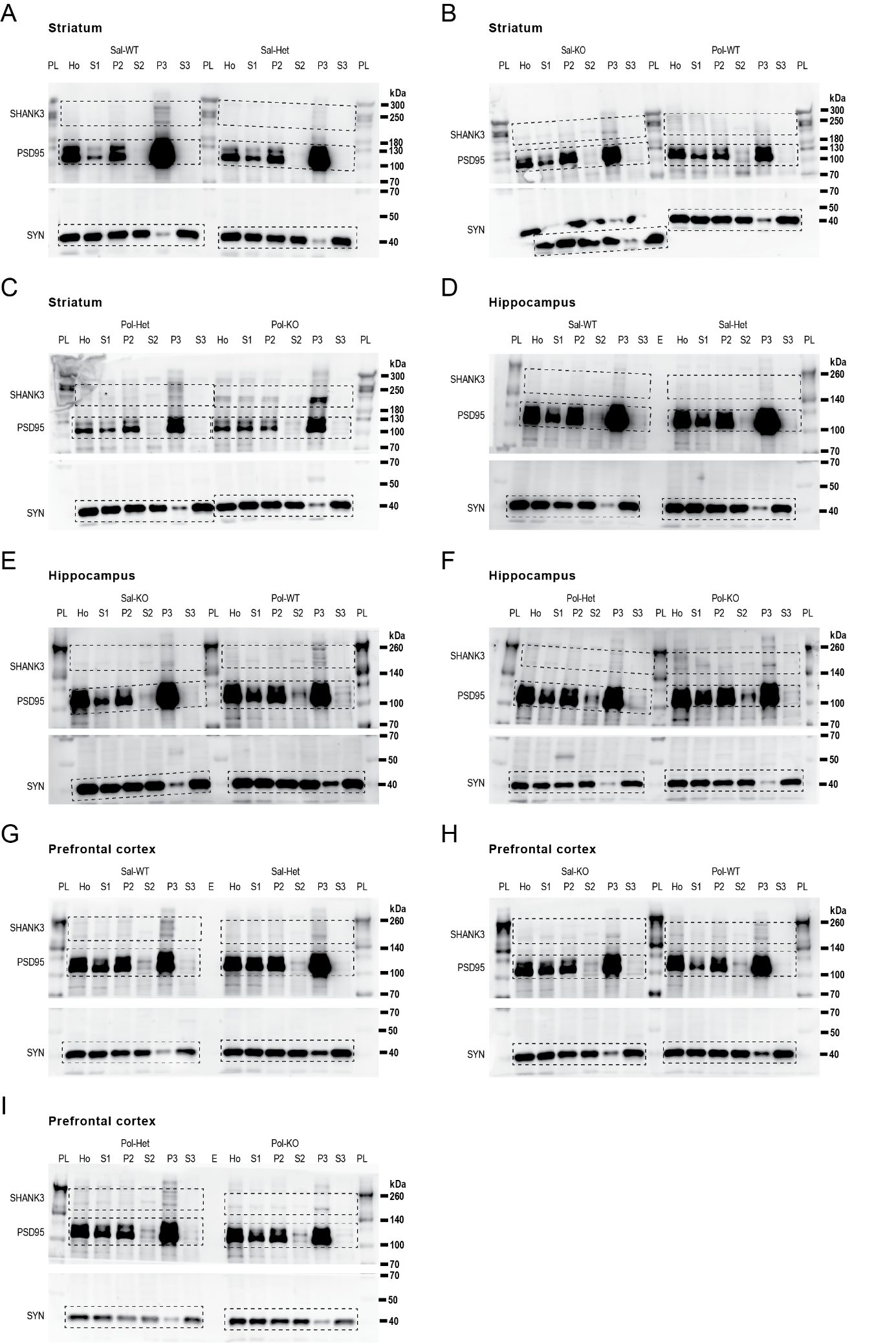


**Suppl. Figure 15. All uncropped Western Blots of the subcellular fractionation of the brain tissue of the offspring of Het dams. A** Uncropped Western Blots of SHANK3, PSD95 (postsynaptic) and SYNAPTOPHYSIN (SYN, presynaptic) – striatum of Sal-WT and Sal-Het offspring. **B** Uncropped Western Blots of SHANK3, PSD95 (postsynaptic) and SYNAPTOPHYSIN (SYN, presynaptic) – striatum of Sal-KO and Pol-WT offspring. **C** Uncropped Western Blots of SHANK3, PSD95 (postsynaptic) and SYNAPTOPHYSIN (SYN, presynaptic) – striatum of Pol-Het and Pol-KO offspring. **D** Uncropped Western Blots of SHANK3, PSD95 (postsynaptic) and SYNAPTOPHYSIN (SYN, presynaptic) – hippocampus of Sal-WT and Sal-Het offspring. **E** Uncropped Western Blots of SHANK3, PSD95 (postsynaptic) and SYNAPTOPHYSIN (SYN, presynaptic) – hippocampus of Sal-KO and Pol-WT offspring. **F** Uncropped Western Blots of SHANK3, PSD95 (postsynaptic) and SYNAPTOPHYSIN (SYN, presynaptic) – hippocampus of Pol-Het and Pol-KO offspring. **G** Uncropped Western Blots of SHANK3, PSD95 (postsynaptic) and SYNAPTOPHYSIN (SYN, presynaptic) – prefrontal cortex of Sal-WT and Sal-Het offspring. **H** Uncropped Western Blots of SHANK3, PSD95 (postsynaptic) and SYNAPTOPHYSIN (SYN, presynaptic) – prefrontal cortex of Sal-KO and Pol-WT offspring. **I** Uncropped Western Blots of SHANK3, PSD95 (postsynaptic) and SYNAPTOPHYSIN (SYN, presynaptic) – prefrontal cortex of Pol-Het and Pol-KO offspring. **A-C** Protein ladder used – Spectra High Range **D-I** Protein ladder used – Spectra Broad Range. **A-I** PL = protein ladder, E = empty lane.

**
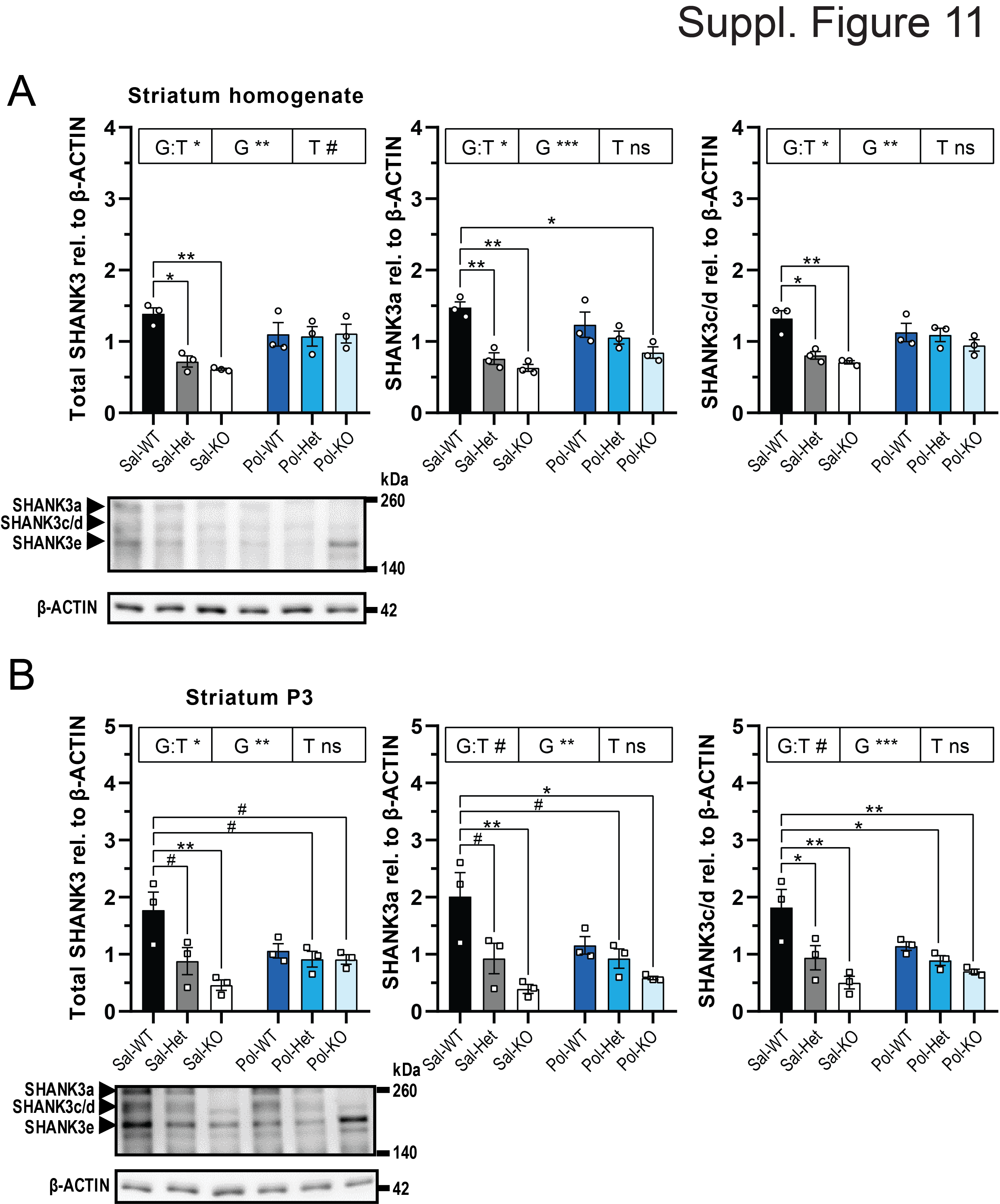
**

**Suppl. Figure 16. SHANK3 expression in striatum of the offspring of Het dams. A** Western Blot analysis for SHANK3 and β-ACTIN in homogenate. Total SHANK3: G:T *p=0.0102, G **p=0.0095, T #p=0.0630, Sal-WT vs Sal-KO **p=0.0058, Sal-WT vs Sal-Het *p=0.0190. SHANK3a: G:T *p=0.0450, G ***p=0.0002, Sal-WT vs Sal-KO **p=0.0011, Sal-WT vs Sal-Het **p=0.0047, Sal-WT vs Pol-KO *p=0.0132. SHANK3c/d: G:T *p=0.0373, G **p=0.0024, Sal-WT vs Sal-KO **p=0.0059, Sal-WT vs Sal-Het *p=0.0224. **B** Western Blot analysis for SHANK3 and β-ACTIN in the P3 fraction. Total SHANK3: G:T *p=0.0258, G **p=0.0055, Sal-WT vs Sal-KO **p=0.0046, Sal-Het vs Sal-WT #p=0.0774, Pol-Het vs Sal-WT #p=0.0994, Pol-KO vs Sal-WT #p=0.0956. SHANK3a: G:T #p=0.0851, G **p=0.0014, Sal-Het vs Sal-WT #p=0.0801, Sal-KO vs Sal-WT **p=0.0042, Pol-Het vs Sal-WT #p=0.0792, Pol-KO vs Sal-WT *p=0.0116. SHANK3c/d: G:T #p=0.0598, G ***p=0.0007, Sal-Het vs Sal-WT *p=0.0456, Sal-KO vs Sal-WT **p=0.0019, Pol-Het vs Sal-WT *p=0.0315, Pol-KO vs Sal-WT **p=0.0076. **A-B** Data were tested for normality with the Shapiro-Wilk test followed by two-way ANOVA with a Bonferroni correction for multiple comparisons. Significance level was set to 0.05 (#<0.10, *p<0.05, **p<0.01, ***p<0.001, ****p<0.0001). Mean ± SEM, n=3. G = Genotype of the offspring; T = Treatment of the mother; G:T = interaction between the two factors; ns = not significant.


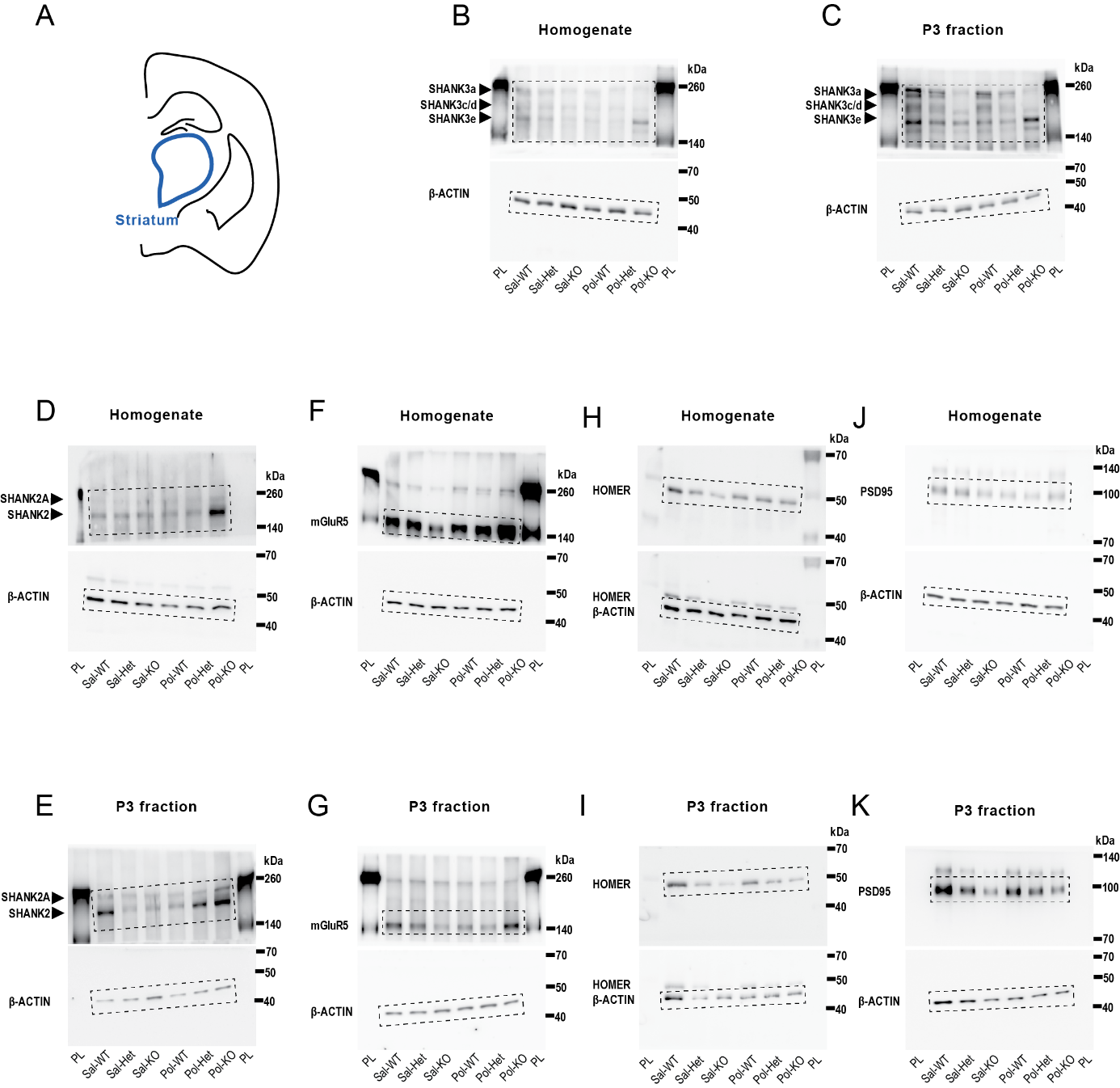


**Suppl. Figure 17. All uncropped Western Blots –striatum of the offspring of Het dams. A** Representative scheme of the mouse striatum. **B+C** Uncropped Western Blots of SHANK3 and β-ACTIN in homogenate (**B**) and in the P3 fraction (**C**). **D+E** Uncropped Western Blots of SHANK2 and β-ACTIN in homogenate (**D**) and in the P3 fraction (**E**). **F+G** Uncropped Western Blots of mGluR5 and β-ACTIN in homogenate (**F**) and in the P3 fraction (**G**). **H+I** Uncropped Western Blots of HOMER and β-ACTIN in homogenate (**H**) and in the P3 fraction (**I**). **J+K** Uncropped Western Blots of PSD95 and β-ACTIN in homogenate (**J**) and in the P3 fraction (**K**). **B-K** On several blots HOMER can be seen next to ß-ACTIN, this is due to the fact that sometimes HOMER was detected first and then the membrane was incubated with ß-ACTIN again over night. PL = protein ladder

**
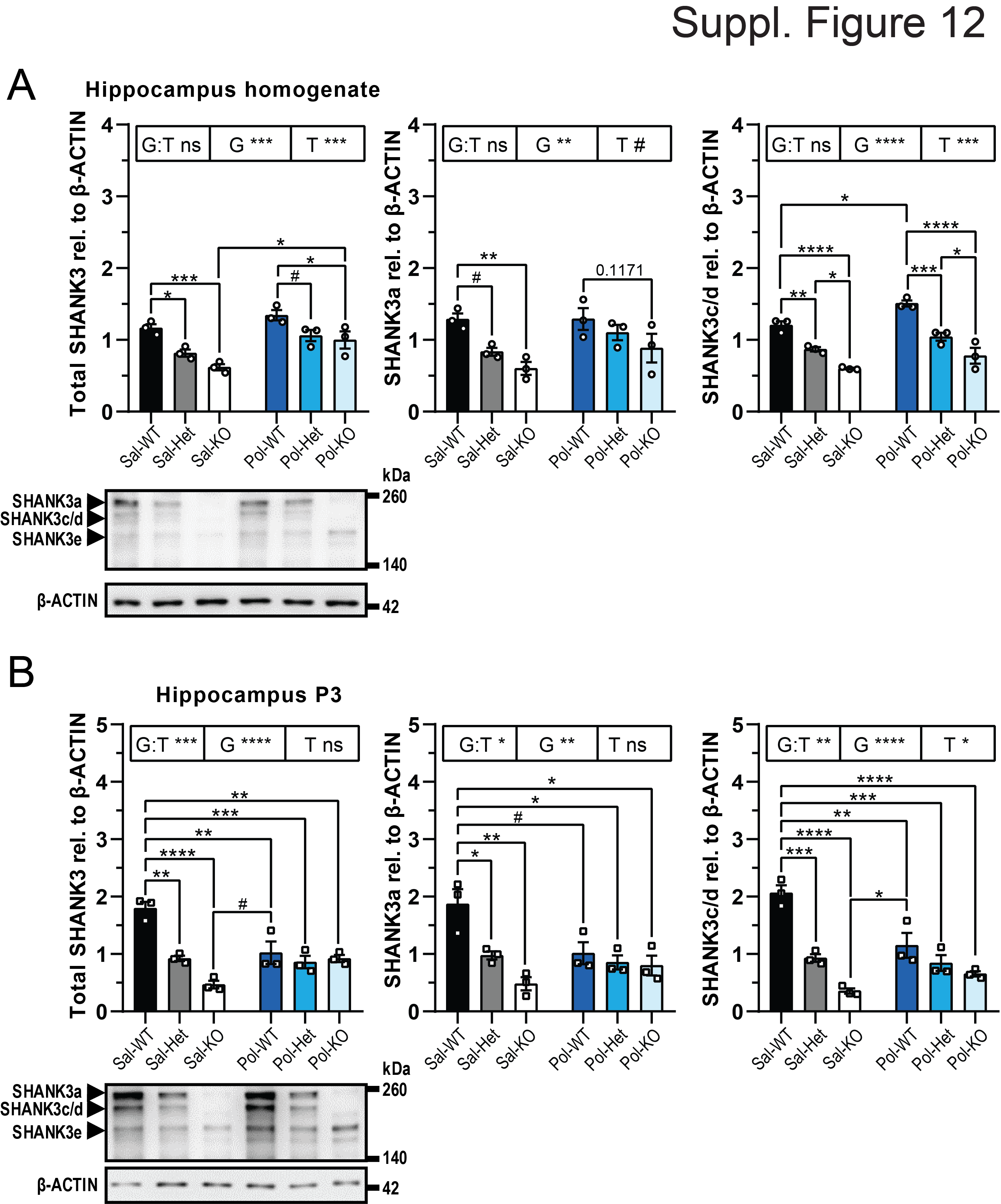
**

**Suppl. Figure 18. SHANK3 expression in hippocampus of the offspring of Het dams. A** Western Blot analysis for SHANK3 and β-ACTIN in homogenate. Total SHANK3: G ***p=0.0002, T ***p=0.0008, Sal-WT vs Sal-KO ***p=0.0006, Pol-WT vs Pol-KO *p=0.0188, Sal-WT vs Sal-Het *p=0.0173, Pol-WT vs Pol-Het #p=0.0565, Pol-KO vs Sal-KO *p=0.0101. SHANK3a: G **p=0.0030, T #p=0.0932, Sal-WT vs Sal-KO **p=0.0062, Sal-WT vs Sal-Het #p=0.0713, Pol-WT vs Pol-KO p=0.1171. SHANK3c/d: G ****p<0.0001, T ***p=0.0007, Sal-WT vs Sal-KO ****p<0.0001, Sal-Het vs Sal-KO *p=0.0228, Pol-WT vs Pol-KO ****p<0.0001, Pol-Het vs Pol-KO *p=0.0252, Sal-WT vs Sal-Het **p=0.0052, Pol-WT vs Pol-Het ***p=0.0004, Pol-WT vs Sal-WT *p=0.0109. **B** Western Blot analysis for SHANK3 and β-ACTIN in the P3 fraction. Total SHANK3: G:T ***p=0.0004, G ****p=<0.0001, Sal-WT vs Sal-Het **p=0.0016, Sal-WT vs Sal-KO ****p<0.0001, Sal-WT vs Pol-WT **p=0.0046, Sal-WT vs Pol-Het ***p=0.0009, Sal-WT vs Pol-KO **p=0.0016, Sal-KO vs Pol-WT #p=0.0582. SHANK3a: G:T *p=0.0135, G **p=0.0014, Sal-WT vs Sal-Het *p=0.0394, Sal-WT vs Sal-KO **p=0.0011, Sal-WT vs Pol-WT #p=0.0523, Sal-WT vs Pol-Het *p=0.0157, Sal-WT vs Pol-KO *p=0.0104. SHANK3c/d: G:T **p=0.0012, G ****p<0.0001, T *p=0.0389, Sal-WT vs Sal-Het ***p=0.0005, Sal-WT vs Sal-KO ****p<0.0001, Sal-WT vs Pol-WT **p=0.0032, Sal-WT vs Pol-Het ***p=0.0002, Sal-WT vs Pol-KO ****p<0.0001, Sal-KO mice vs Pol-WT *p=0.0102. **A-B** Data were tested for normality with the Shapiro-Wilk test followed by two-way ANOVA with a Bonferroni correction for multiple comparisons. Significance level was set to 0.05 (#<0.10, *p<0.05, **p<0.01, ***p<0.001, ****p<0.0001). Mean ± SEM, n=3. G = Genotype of the offspring; T = Treatment of the mother; G:T = interaction between the two factors; ns = not significant.


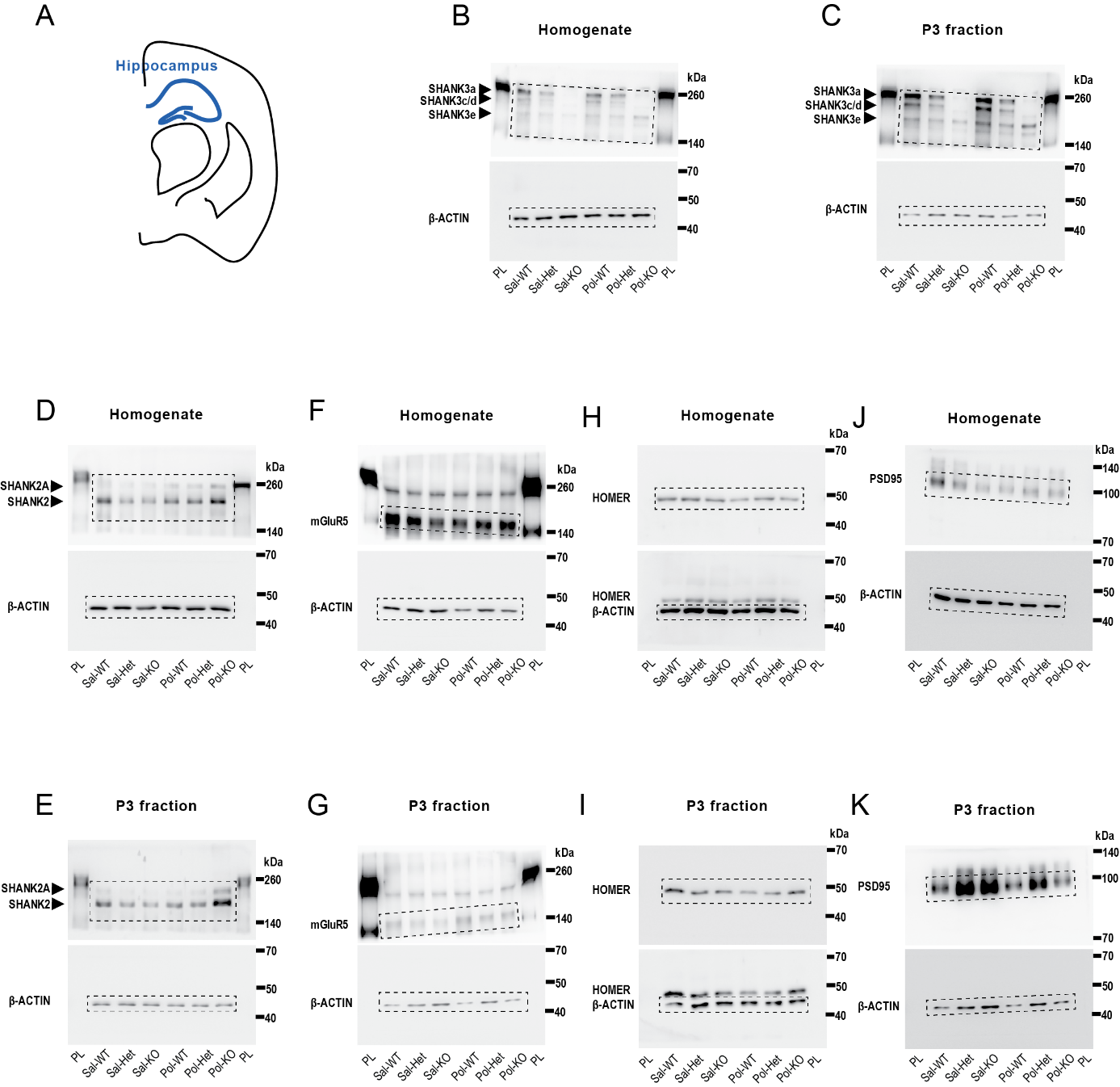


**Suppl. Figure 19. All uncropped Western Blots – hippocampus of the offspring of Het dams. A** Representative scheme of the mouse hippocampus. **B+C** Uncropped Western Blots of SHANK3 and β-ACTIN in homogenate (**B**) and in the P3 fraction (**C**). **D+E** Uncropped Western Blots of SHANK2 and β-ACTIN in homogenate (**D**) and in the P3 fraction (**E**). **F+G** Uncropped Western Blots of mGluR5 and β-ACTIN in homogenate (**F**) and in the P3 fraction (**G**). **H+I** Uncropped Western Blots of HOMER and β-ACTIN in homogenate (**H**) and in the P3 fraction (**I**). **J+K** Uncropped Western Blots of PSD95 and β-ACTIN in homogenate (**J**) and in the P3 fraction (**K**). **B-K** On several blots HOMER can be seen next to ß-ACTIN, this is due to the fact that sometimes HOMER was detected first and then the membrane was incubated with ß-ACTIN again over night. PL = protein ladder

**
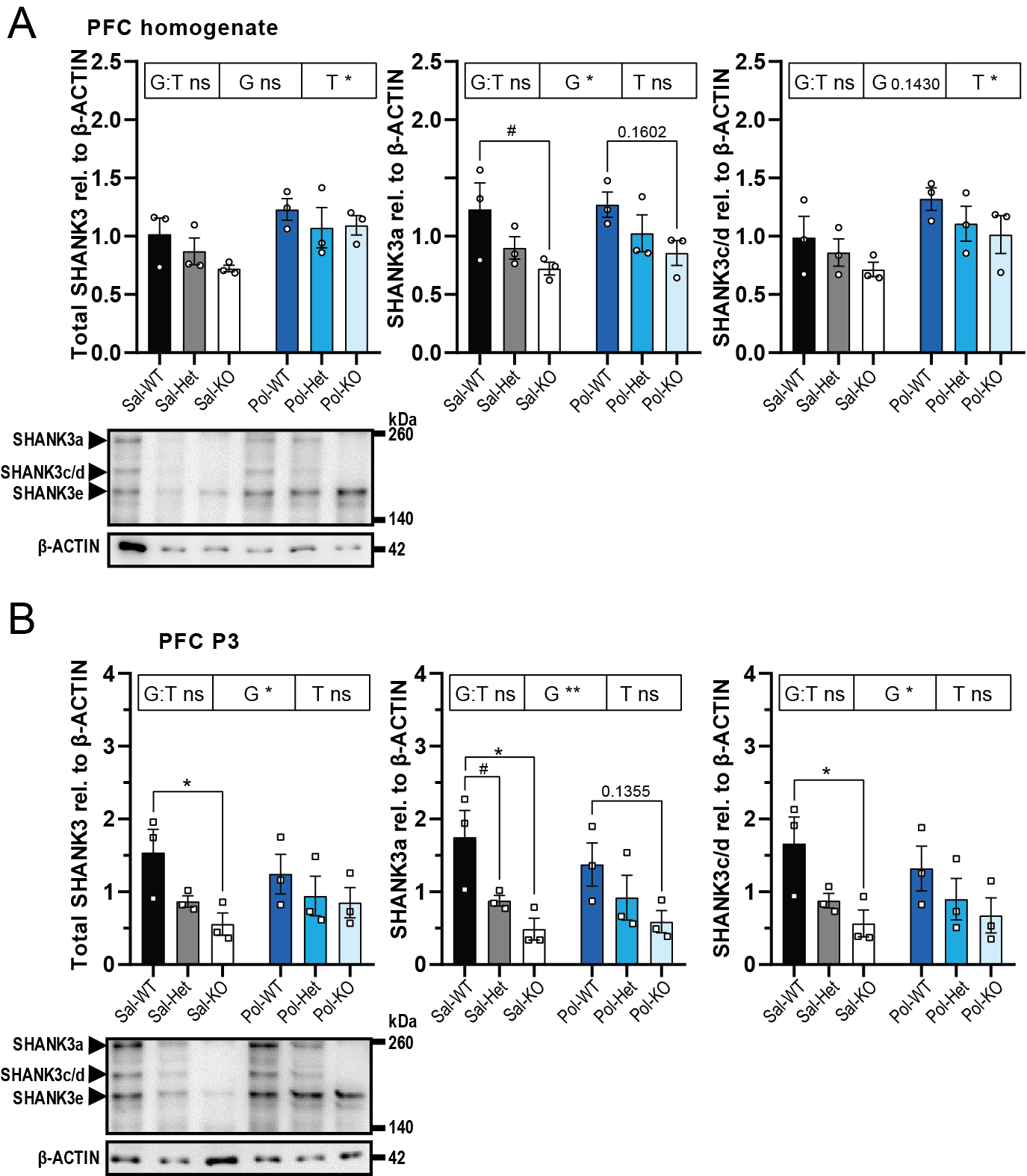
**

**Suppl. Figure 20. SHANK3 expression in PFC of the offspring of Het dams. A** Western Blot analysis for SHANK3 and β-ACTIN in homogenate. Total SHANK3: T *p=0.0161. SHANK3a: G *p=0.0175, Sal-WT vs Sal-KO #p=0.0675, Pol-WT vs Pol-KO p=0.1602. SHANK3c/d: G p=0.1430, T *p=0.0208. **B** Western Blot analysis for SHANK3 and β-ACTIN in the P3 fraction. Total SHANK3: G *p=0.0322, Sal-WT vs Sal-KO *p=0.0338. SHANK3a: G **p=0.0047, Sal-WT vs Sal-KO *p=0.0114, Sal-WT vs Sal-Het #p=0.0889, Pol-WT vs Pol-KO p=0.1355. SHANK3c/d: G *p=0.0174, Sal-WT vs Sal-KO *p=0.0360. **A-B** Data were tested for normality with the Shapiro-Wilk test followed by two-way ANOVA with a Bonferroni correction for multiple comparisons. Significance level was set to 0.05 (#<0.10, *p<0.05, **p<0.01, ***p<0.001, ****p<0.0001). Mean ± SEM, n=3. G = Genotype of the offspring; T = Treatment of the mother; G:T = interaction between the two factors; ns = not significant.

**
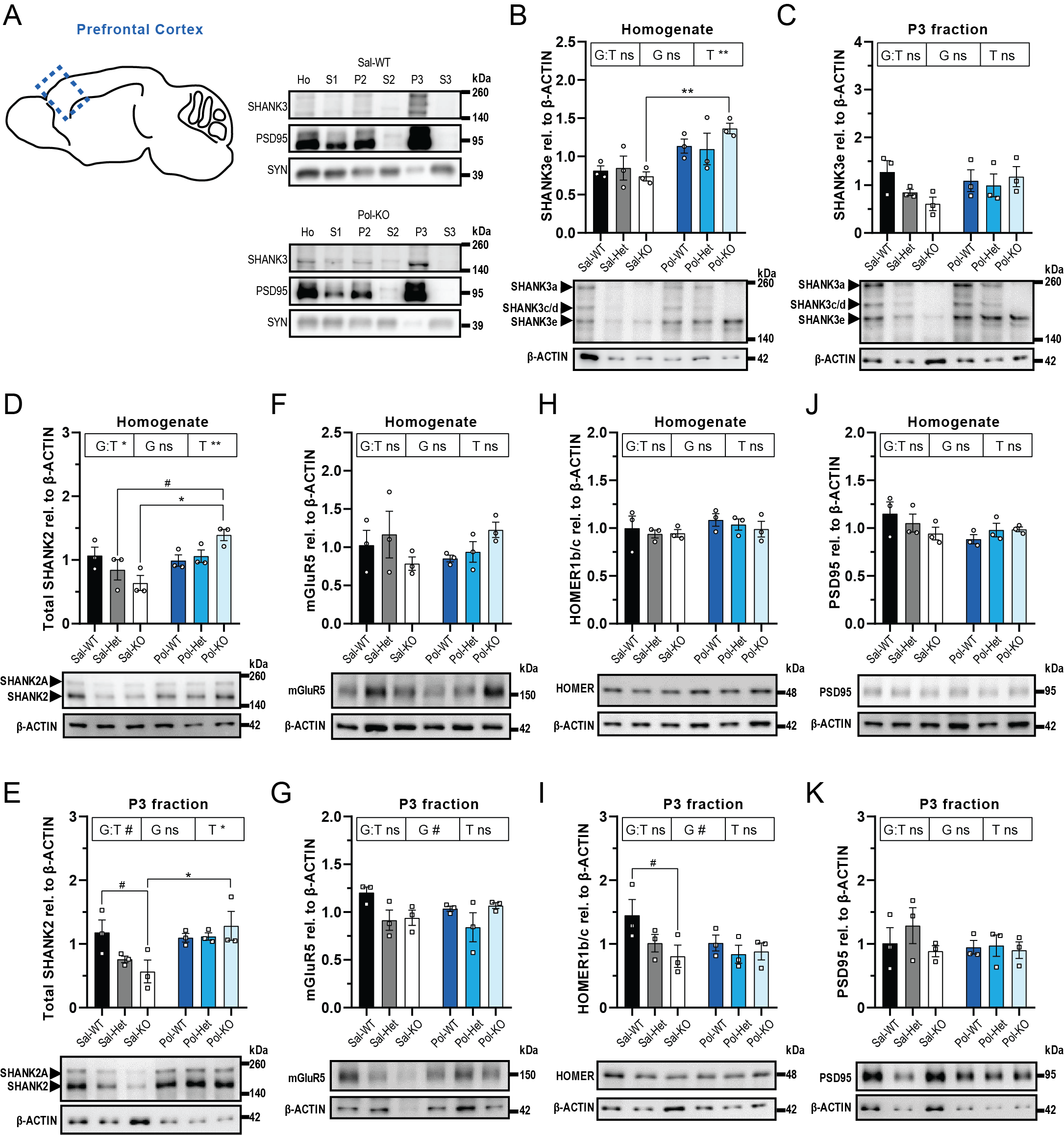
**

**Suppl. Figure 21. Synaptic changes in PFC of the offspring of Het dams. A** Fractionation of the PFC. Western Blots of Sal-WT and Pol-KO are shown for SHANK3, PSD95 (postsynaptic) and SYNAPTOPHYSIN (SYN, presynaptic). **B** Western Blot analysis for SHANK3 and β-ACTIN of PFC homogenate. The SHANK3e isoform was analyzed. T **p=0.0017, Pol-KO vs Sal-KO **p=0.0100. **C** Western Blot analysis for SHANK3 and β-ACTIN of PFC P3 fraction. The SHANK3e isoform was analyzed. **D** Western Blot analysis for SHANK2 and β-ACTIN of PFC homogenate. The total SHANK2 was analyzed. G:T *p=0.0127, T **p=0.0094, Sal-KO vs Pol-KO *p=0.0104, Sal-Het vs Pol-KO #p=0.0988. **E** Western Blot analysis for SHANK2 and β-ACTIN of PFC P3 fraction. The total SHANK2 was analyzed. G:T #p=0.0591, T *p=0.0186, Sal-KO vs pol-KO *p=0.0158, Sal-WT vs Sal-KO #p=0.0982. **F** Western Blot analysis for mGluR5 and β-ACTIN of PFC homogenate. **G** Western Blot analysis for mGluR5 and β-ACTIN of PFC P3 fraction. G #p=0.0516. **H** Western Blot analysis for HOMER1b/c and β-ACTIN of PFC homogenate. **I** Western Blot analysis for HOMER1b/c and β-ACTIN of PFC P3 fraction. G #p=0.0852, Sal-KO vs Sal-WT #p=0.0530. **J** Western Blot analysis for PSD95 and β-ACTIN of PFC homogenate. **K** Western Blot analysis for PSD95 and β-ACTIN of PFC P3 fraction. **B-K** Data were tested for normality with the Shapiro-Wilk test followed by two-way ANOVA with a Bonferroni correction for multiple comparisons. Significance level was set to 0.05 (#<0.10, *p<0.05, **p<0.01, ***p<0.001, ****p<0.0001). Mean ± SEM, n=3. G = Genotype of the offspring; T = Treatment of the mother; G:T = interaction between the two factors; ns = not significant.


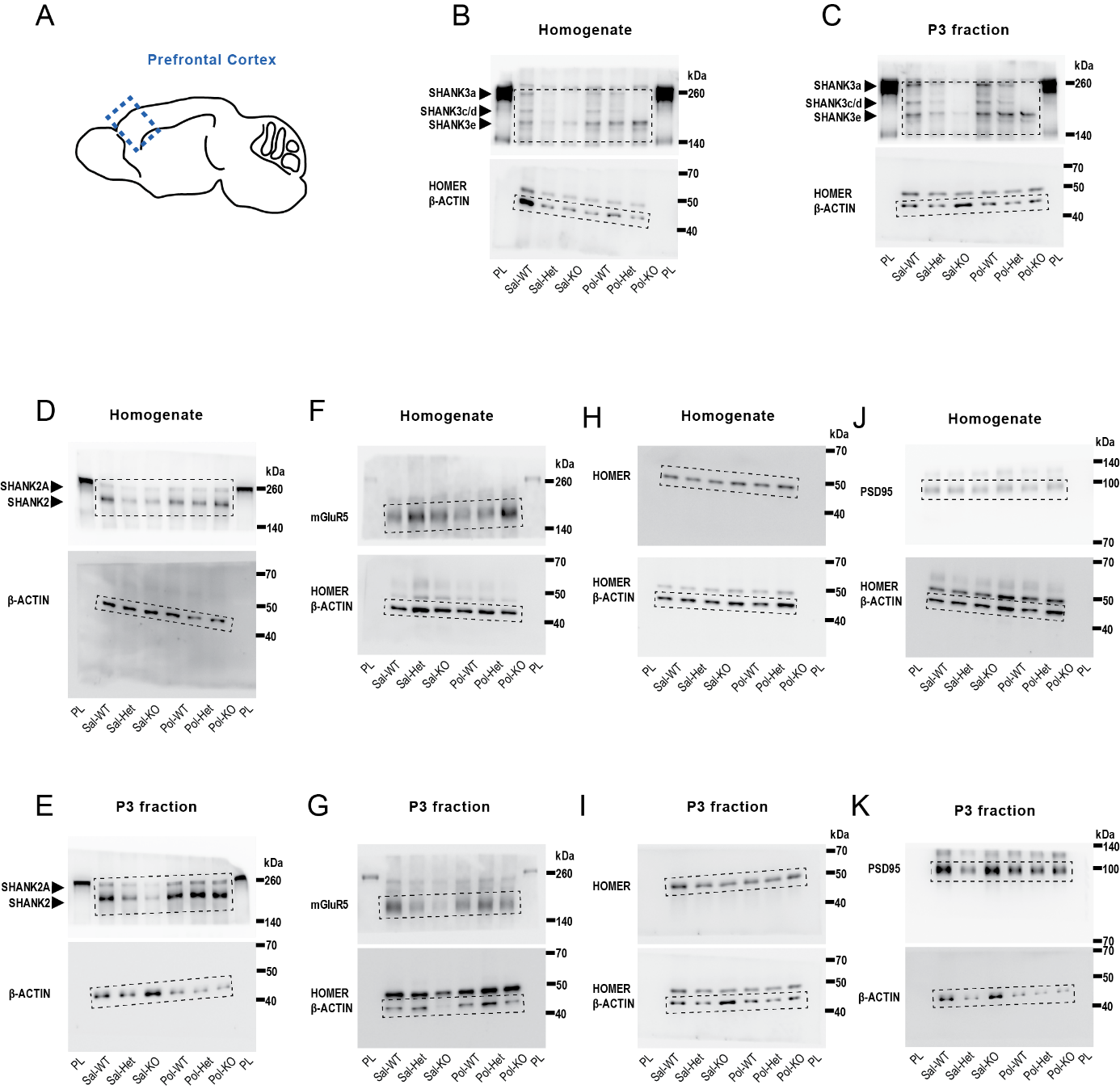


**Suppl. Figure 22. All uncropped Western Blots – prefrontal cortex of the offspring of Het dams. A** Representative scheme of the mouse prefrontal cortex. **B+C** Uncropped Western Blots of SHANK3 and β-ACTIN in homogenate (**B**) and in the P3 fraction (**C**). **D+E** Uncropped Western Blots of SHANK2 and β-ACTIN in homogenate (**D**) and in the P3 fraction (**E**). **F+G** Uncropped Western Blots of mGluR5 and β-ACTIN in homogenate (**F**) and in the P3 fraction (**G**). **H+I** Uncropped Western Blots of HOMER and β-ACTIN in homogenate (**H**) and in the P3 fraction (**I**). **J+K** Uncropped Western Blots of PSD95 and β-ACTIN in homogenate (**J**) and in the P3 fraction (**K**). **B-K** On several blots HOMER can be seen next to ß-ACTIN, this is due to the fact that sometimes HOMER was detected first and then the membrane was incubated with ß-ACTIN again over night. PL = protein ladder
